# EnviroSpect-guided ConvMixer–ViT framework for environment-robust potato leaf disease detection

**DOI:** 10.3389/fpls.2026.1886452

**Published:** 2026-08-21

**Authors:** Abida Sharif, Mudassir Khalil, Muhammad Zaheer Sajid, Reem Alshenaifi, Jarrar Amjad

**Affiliations:** 1 Shien-Ming Wu School of Intelligent Engineering, South China University of Technology, Guangzhou, China; 2 Computer Engineering Department, Bahauddin Zakariya University, Multan, Pakistan; 3 Department of Electrical and Computer Engineering, George Mason University, Fairfax, VA, United States; 4 Department of Information Technology, College of Computer and Information Sciences, Majmaah University, Al Majmaah, Saudi Arabia; 5 Department of Computer Science, Kansas State University, Manhattan, KS, United States

**Keywords:** agricultural AI, deep learning, feature fusion, image classification, prototypical network

## Abstract

Potato is one of the world’s most important staple crops, yet its productivity is persistently compromised by foliar diseases particularly early blight (*Alternaria solani*) and late blight (*Phytophthora infestans*) which together account for substantial annual yield losses. Conventional visual diagnosis by agronomists is laborintensive, subjective, and ill-suited to large-scale deployment, while prevailing deep learning solutions tend to degrade under real-world variability in illumination, background, and leaf orientation, and typically require large, annotated datasets that are rarely available in field conditions. To address these limitations, we propose a hybrid deep learning framework that couples ConvMixer, for localized lesionscale feature extraction, with a Vision Transformer (ViT), for modeling long-range spatial dependencies, and aggregates their representations through an elementwise ensemble. The pipeline is preceded by EnviroSpect, a novel preprocessing scheme that independently remaps hue, enhances saturation, and normalizes intensity in a custom hue–saturation–intensity (HSI)-based color space to preserve disease-discriminative cues under heterogeneous imaging conditions. Classification is performed by a prototypical-network-based head, which leverages class prototypes computed from training features to produce similarity-based decisions that complement standard softmax classification. The framework was trained and evaluated on three datasets: PlantVillage, Potato Plants, and a custom field-acquired AgriPlant set, together comprising 5,304 images across three disease classes. The proposed model attains classification accuracies of 99% on PlantVillage and 98% on Potato Plants, with a macro F1-score of 99.02%, precision of 99.20%, and recall of 98.85%, consistently outperforming individual ConvMixer, ViT, ResNet18, MobileNetV2, and InceptionV3 baselines. Ablation and statistical significance analyses (*p<* 0.05) confirm that each component — the hybrid backbone, EnviroSpect preprocessing, and the prototypical network head contribute meaningfully to overall performance, with EnviroSpect providing the largest single improvement. Importantly, the model achieves these results with only 8.9 M parameters and 19 ms per-image graphics processing unit (GPU) inference, striking a favorable accuracy– efficiency balance for deployment on resource-constrained edge devices. The proposed framework achieves a strong accuracy–efficiency balance for potato leaf disease detection and offers a transferable foundation that could be extended toward scalable, environment-robust plant-disease monitoring in other crops and agricultural scenarios.

## 1 Introduction

Potato is the fourth most important staple food crop globally after rice, wheat, and maize only because of its high content of nutrition, versatility, and tolerance to varied environmental conditions. As one of the world’s indispensable non-cereal crops, potato is an important source of food security and a key driver of economic growth (Qu et al., 2005; Wang et al., 2023). However, potato production faces constant threats from several diseases; the most widespread and destructive diseases incorporate early blight (*Alternaria solani*) and late blight. These diseases keep yields lower than they should be and affect not only small-scale farmers but also the largest agricultural scale farmers (Kirk, 2015). Both early and late blight produce similar symptoms, in terms of scorching leaves and tubers. Both diseases are often poorly identified; management is usually not done on time. Efficient detection and assessment of the severity of these diseases is an important attribute of successful crop management (Park et al., 2023). Traditional methods of monitoring disease severity in potato crops relied on agricultural technicians, experts, or farmers who visually estimated the percent infection area of the plants. This, though widely practiced, is highly subjective and calls for so much time, labor, and inconsistencies (Granja et al., 2013). Therefore, with increasing demands for efficient, scalable, and more accurate methods for classification, diagnosis, and disease severity assessment in potato crops, this is inevitable. Fortunately, with the advancement of image processing and artificial intelligence (AI), there are solutions to these present challenges. These new methods provide automatic identification and categorization of the disease level based on the infected plants’ appearance. They do this by using color, texture, and pattern parameters of the area taken from the photograph. Deep learning models have driven significant advances in plant disease classification and detection over the past few years. Different architectures of convolutional neural network (CNN), such as VGG (Simonyan and Zisserman, 2014), ResNet (He et al., 2016), and MobileNet (Howard et al., 2017), are very active in classifying early blight and late blight disease that attacks potato crops. These will be more useful in large-scale agricultural applications where the manual inspection is rather impractical. For example, VGG makes use of deep architecture for large-scale image recognition, whereas ResNet proposed residual links to counteract the vanishing gradient problem in deeper networks, which improved accuracy in disease classification (Simonyan and Zisserman, 2014; He et al., 2016). Similarly, MobileNet provides a lightweight network that is both highly efficient and very accurate, rendering it best suited for real-time disease monitoring in resource-limited environments (Howard et al., 2017). Several studies have successfully applied these models for plant disease classification. For instance, Yin et al. suggested a deep learning model named DISE-Net for the classification of severity level of maize leaf spot disease that was 97.12% accurate in classification (Yin et al., 2022). This figure, however, is difficult to interpret in isolation: it is reported on maize rather than potato, and the study does not benchmark the model against a common CNN baseline on the same data, so the share of the gain attributable to the proposed architecture rather than to the dataset itself cannot be separated. The relevance of DISE-Net to the present work therefore lies in its architectural strategy of coupling lesion-scale feature extraction with severity-aware classification, rather than in the reported accuracy value. In addition, some multi-stage approaches integrating more than one deep learning task have demonstrated immense potential for increasing the accuracy in the classification of disease for multi-diseaseinfected crops. Li et al. proposed a method using an integrated approach that combines a classification and detection task. This has been able to classify potato foliage diseases in an environment with complex backgrounds and variable conditions (Li et al., 2022). The goal of the research is to develop a deep-learning model that can automatically classify potato leaf diseases (PLD). The paper contributes to sustainable agriculture by addressing potato crop health, essential for both nutrition and economic stability in farming communities. The ensemble model has been created using the ConvMixer and Vision Transformer architecture. These visual symptoms and patterns of the leaves, with the help of images, have classified PLD effectively while training the ensembled model. Disease features on the blight have been identified by agricultural experts in order to inform the training process. This study is critically relevant because it proposes a new way of classifying PLD, which is highly relevant for field applications in agricultural disease management. Summarized results on PLD that are tabulated in **Table 1**.

**Table 1:** Potato leaf findings categorized by severity level.

Serial No.	Disease name	Disease explanation
1	Potato Early Blight	*Alternaria solani* leaves small, dark brown, circular spots. These increase in size, thus hugely defoliating and reducing yields.
2	Potato Healthy	Fresh green leaves are a sign of healthy potato plants that are free of all forms of spotting and discoloration and are a good development under disease-free conditions.
3	Potato Late Blight	*Phytophthora infestans* has already been identified to be the cause of the foremost potato syndrome, Potato Late Blight. It shows water-saturated cuts that develop successively on leaves and stems; it has a prompt decay that might contribute to crop loss.

Source: compiled by the authors. The descriptions summarise the characteristic visual symptoms of each class as observed in the potato leaf images of the three datasets used in this study, and are provided to define the three target classes rather than to report new experimental findings.

### 1.1 Research study motivation

Agricultural diseases, especially in expensive crop products such as potatoes, are a significant threat to world food security, the output of crops, and the economy. In the case of these diseases, early and late blight are the most powerful and dangerous ones, and they spread to a large extent, causing considerable losses to small- and large-scale farmers throughout the globe (Park et al., 2023). The diagnosis of diseases by specialists, which is the traditional method, may be very time-consuming and exhausting, as well as subjective and problematic in terms of scalability (Granja et al., 2013). Besides, their precision is diminished because these environmental conditions, such as lighting and background textures, are inconsistent. Although the use of deep learning and computer vision has opened new possibilities for the automatic detection of diseases in plants, current models hardly generalize different field conditions, confuse disease stages that look similar, and require high computational power, which is not available in agricultural areas that are poor in resources. The magnitude of this generalization gap is documented within the literature itself: a hybrid CNN–ViT model that reaches 98.15% on the controlled PlantVillage benchmark falls to 85.06% on uncontrolled field imagery (Sinamenye et al., 2025), indicating that accuracies obtained under laboratory acquisition are weak predictors of field behaviour. These issues show the urgent need for highly accurate, resource-efficient, and flexible disease detection system to work under the real world variation with minimal amount of labeled data. Based on these issues the paper proposed a novel hybrid deep learning model consists of ConvMixer and Vision Transformer (ViT) with novel preprocessing method named EnviroSpect and a prototype network based classification head. The purpose of the paper is to increase the accuracy and robustness of the disease detection system with application case study in real agricultural environment.

From the above discussion, a clear knowledge gap can be identified. First, most existing PLD detection models rely on either local convolutional features or global attention features alone, which limits their ability to capture the fine-grained lesion patterns and the broader contextual cues that are simultaneously required to distinguish visually similar disease stages such as early and late blight. Second, the performance of many models degrades under real field variability in illumination, background, and leaf orientation, because standard preprocessing methods (for example contrast limited adaptive histogram equalization (CLAHE), Retinex, and red–green–blue (RGB)-to-hue– saturation–value (HSV)) are not specifically designed to preserve disease-discriminative color and texture cues under heterogeneous imaging conditions. Third, several highaccuracy models are computationally demanding, which restricts their deployment on the resource-constrained devices typically available in agricultural settings, while lightweight models often sacrifice accuracy (Kasana and Rathore, 2024). To address these gaps, this study is guided by the following objectives: (i) to design a hybrid backbone that jointly exploits local features from ConvMixer and global dependencies from a Vision Transformer for more discriminative representation of disease symptoms; (ii) to develop the EnviroSpect preprocessing scheme that remaps hue, enhances saturation, and normalizes intensity in a custom HSI color space in order to retain disease-relevant cues under variable environmental conditions; (iii) to incorporate a prototypical network classification head that forms class prototypes and assigns samples by similarity, yielding more stable decision boundaries; and (iv) to achieve this while maintaining a favorable accuracy–efficiency balance suitable for deployment on resource-constrained edge devices.

### 1.2 Research study contribution

The study that follows significantly advances the categorization of potato illnesses:

- This research presents an innovative hybrid deep learning model that integrates ConvMixer for local feature extraction and Vision Transformer (ViT) for capturing global dependencies. The complementary strengths of these models significantly enhance the classification accuracy of PLD, including early blight, late blight, and healthy conditions.
- A novel preprocessing technique, EnviroSpect, is introduced to address environmental variability in agricultural settings. By normalizing intensity, hue, and saturation, this method is designed to support more consistent feature extraction across diverse lighting, weather, and background conditions.
- The study incorporates a prototypical network classification head that computes class prototypes from the fused ConvMixer–ViT features and classifies new samples based on Euclidean distance to these prototypes. This similarity-based decision mechanism produces more stable class boundaries and complements the hybrid feature extractor.
- The proposed ConvMixer-ViT model achieves outstanding performance, with an accuracy of 98% on the Potato Plants dataset and 99% on the PlantVillage dataset. These results surpass existing models, highlighting the model’s robustness and accuracy in disease classification.
- Optimized for computational efficiency, the model is designed to operate in resourceconstrained environments. Its ability to balance high accuracy with minimal computational demands makes it well-suited for real-time disease monitoring in agricultural settings.
- The scalability of the framework enables easy adaptation to other crops and agricultural challenges, paving the way for future research in multi-crop disease detection and the broader application of sustainable farming practices.

The novelty of this work lies not in the individual components but in their targeted integration and in the field-robust design of EnviroSpect. Each component is chosen for a complementary reason: ConvMixer for compact, parameter-efficient local feature extraction (lighter than VGG or ResNet); the Vision Transformer for the long-range dependencies needed to disambiguate visually similar early and late blight; and the prototypical network head, over a plain softmax, for more stable class boundaries and a path toward low-shot operation. Unlike CLAHE, Retinex, and RGB-to-HSV, which enhance contrast, normalize illumination, or separate chrominance in isolation, EnviroSpect operates in a custom HSI space and jointly remaps hue toward the vegetation spectrum, enhances saturation, and normalizes intensity to keep lesion cues discriminable under heterogeneous conditions.

## 2 Related work

PLD identification and classification is a widespread research area because of decline in potato yield and quality. Different machine learning and deep learning algorithms are investigated by the researchers for improving the accuracy of potato crop leaf disease identification and classification. These are diseases caused by fungi, which, if not easily detected at an early stage, can decrease the potato yield to a very high level. Here, the critical review of the research concerning different models, approaches, and performance measures for detecting PLD is presented.

Khalifa et al. (2021) introduced a CNN-based method for PLD classification targeting early blight and late blight. The suggested method was a 14-layer CNN architecture, where the main convolutional layer was repeated twice for feature extraction purposes, and the last two layers were fully connected for classification purposes. The authors augmented their data using different data augmentation methods; as a result, their dataset increased from 1,722 images to 9,822 images, which enhanced the effectiveness of their classifier. The CNN model obtained an average testing accuracy of 98% across more than six performance metrics. The authors attribute this performance to two main convolutional blocks operating at different window sizes, giving receptive fields at two scales so that small early-blight lesions and the larger diffuse regions typical of late blight are represented within the same feature hierarchy, with the augmentation of the training set from 1,722 to 9,822 images accounting for the remainder. The improvement is established against accuracies previously published for the same task, however, rather than against baselines retrained under matched conditions, and since those results used different dataset partitions the margin cannot be attributed to the architecture alone. A further limitation is that the 9,822 images were expanded from only 1,722 originals, so the reported 98% is sensitive to whether the split was performed before or after augmentation. Unless the partition was made at the level of source images, the score reflects accuracy on augmented variants of leaves already seen in training rather than generalisation to unseen field conditions. Kumar and Patel (2023) presented an HDLCNN for identifying and classifying the diseases of potato leaves. The approach initiates with a pre-processing step to filter off the noisy portion of images using Median Filtering. Then, this approach employed IFLBP for the feature extraction process. HDL CNN classifies the leaf diseases and helps in recommending treatment methodologies with the support of Decision Support Systems to farmers. It provides superior results than the general methodology, VGG-INCEP, and Random Forest by 4–6% in accuracy, precision, recall, and F-score. The proposed methodology was implemented in MATLAB-Simulink software that showed improvement in the performance of a classifier with high accuracy. This is one of the few studies in the group that reports a margin against named baselines rather than an absolute score alone, which makes the 4–6% gain interpretable. The gain is nonetheless obtained from hand-engineered IFLBP descriptors feeding a conventional CNN, so the pipeline depends on a fixed texture operator that must be retuned when lesion appearance changes with illumination or leaf orientation. Tiwari et al. (2020) implemented transfer learning by utilizing the VGG19 model on a potato leaf dataset infested with diseases. In this regard, transfer learning, particularly the fine-tuning of pre-trained models such as VGG19, has been found to perform extremely well in cases where the dataset may be small or domain-specific. Their experiments showed that logistic regression gave the highest accuracy of 97.8% when applied on the extracted features. This is in respect to fine-tuning pre-trained models for performance enhancement in disease detection systems.

Suttapakti and Bunpeng (2019) proposed another approach for the detection of PLD with a key focus on exploring distinct color and texture features. According to their technique, they converted the images into Lab color space and performed separation between the background and the diseased region through k-means clustering. They performed the extraction of maximum-minimum color difference features which were combined with the texture features to increase the accuracy in classification. The choice of the Lab space is the methodologically significant element, since it separates lightness from the two chromatic channels and therefore allows the maximum-minimum colour difference descriptor to respond to lesion pigmentation with reduced sensitivity to overall illumination level. The reported gain is stated relative to baseline approaches that are not individually identified, and no per-class figures are given, so the improvement cannot be attributed to the colour descriptor as distinct from the k-means segmentation stage that precedes it. Rashid et al. (2021) suggested a multilevel deep learning architecture for PLD classification. YOLOv5 segmentation technique was applied in the first level to extract potato leaves from an image. In the second level, a novel deep learning approach used a CNN to detect early and late blight in potato leaves. The model introduced in the paper was also trained and tested using the PlantVillage dataset with an accuracy of 99.75%. Their model also surpassed existing state-of-the-art methods as it provides higher accuracy with less computational cost. The 99.75% is, however, obtained entirely on PlantVillage, whose images are captured against uniform laboratory backgrounds. The YOLOv5 stage exists precisely to remove background before classification, which indicates that the pipeline treats background variability as something to be segmented away rather than something the classifier itself must tolerate. Performance under the cluttered soil, straw and mixed-canopy backgrounds encountered in the field is not reported. This multilevel deep learning model offers an end-to-end solution for potato disease detection by combining existing state-of-the-art segmentation and classification methods. Sharma et al. (2021) presented a method comprising some steps involving image processing and machine learning (ML) algorithms for diseased potato leaf image classification. The noisy image was filtered using the Gaussian filter, followed by k-means clustering to segment the region of interest. The various machine learning algorithms tested are support vector machine (SVM), k-nearest neighbors (KNN), Na¨ıve Bayes, and Decision Tree. Among them, the highest accuracy obtained was from the SVM model, which is 92.9%. Therefore, this study illustrates the feasibility of applying traditional algorithms in machine learning along with image processing techniques for PLD classification. The 92.9% obtained by the best classifier is roughly six percentage points below the CNN-based studies reviewed above, and the gap is instructive: the k-means segmentation step that precedes classification depends on colour separability, which degrades exactly under the variable illumination that field imagery introduces. This marks the practical ceiling of handcrafted pipelines and motivates the learned representations used in later work.

Ghosh et al. (2023) conducted a large-scale comparison of three top-performing CNN models-VGG19, DenseNet121, and ResNet50—for PLD detection. Data augmentation was also employed to enhance the generalization ability of their model. The three backbones were assessed on accuracy, precision, recall and F1-score as well as computational efficiency, with VGG19 ranked first and DenseNet121 and ResNet50 close behind. The small margins are the informative part: architectures differing substantially in depth, parameter count and connectivity converge to comparable accuracy, indicating that the ceiling is imposed by the dataset rather than by backbone capacity. The study reports which model ranks highest but does not isolate the mechanism, since no ablation separates the augmentation policy from the architectures, and no common training budget is specified. Radwan et al. (2024) compared several machine learning models, including logistic regression, gradient boosting, multilayer perceptron (MLP), and SVM for the prediction of PLD based on a dataset of weather conditions, while feature selection techniques included the binary Greylag Goose Optimization (bGGO) for the optimization of model performance. Feature selection in the MLP model resulted in an accuracy of 98.3%, proving feature selection to be an important process for enhancing ML models in their accuracy. This work further proved that features related to weather may be used for disease prediction, hence forms a new dimension of integrating environmental factors with potato disease detection. Singh and Kaur (2021) applied K-means clustering for image segmentation and multi-class SVM to classify PLD using the PlantVillage dataset. Their work reached an accuracy of 95.99%, hence showing how well simple segmentation techniques collaborated with good classifiers like SVM. This kind of work proved that conventional methods could give high accuracy in plant disease detection by just combining them effectively.

Chen et al. (2022) presented a lightweight CNN model based on MobileNet-V2, called MobOca Net, for PLD recognition. They incorporated the attention mechanism to enhance learning capability due to minute lesion features. The proposed model achieved an accuracy of 97.73% on the PlantVillage dataset, where most of them attained either higher accuracy or speed but not both simultaneously. This trade-off is central for deployable models: MobOca Net accepts roughly two points below the strongest PlantVillage results in exchange for a MobileNet-V2 footprint. The comparison is drawn against general-purpose backbones rather than other lightweight disease-specific models, however, so the contribution of the attention mechanism is not isolated from that of the compact backbone. It justifies the capability of the lightweight CNN architectures in real-world applications related to agriculture, where computational resources are severely limited. Hasan et al. (2021) compared three deep CNN models for PLD classification, namely AlexNet, ResNet, and GoogLeNet. The authors prepared their own dataset by augmentation through rotation and scaling and then divided it into training and testing sets. ResNet performed best among the three discussed models and provided the best accuracy, precision, recall, and F1-score. The three backbones embody distinct design principles-AlexNet a shallow stack of wide convolutions, GoogLeNet parallel multi-scale inception blocks, and ResNet identity shortcuts permitting greater depth—so the advantage of ResNet is consistent with depth being the limiting factor when lesion features must be separated from healthy tissue at several scales. The result is reported as a ranking without per-model figures, however, and the dataset was assembled by the authors rather than drawn from a public benchmark, so neither the margin nor its reproducibility can be assessed.Rozaqi and Sunyoto (2020) conducted a study regarding the use of CNN architectures for the identification of early blight and late blight diseases that affect potato leaves. The dataset was divided into training and validation sets. The accuracy achieved in the training data using the CNN model was 97%, while in the validation dataset, it was 92%. From this work, it can be ascertained that CNN models, when supplemented, are indeed capable of achieving high accuracies, hence making them a very powerful tool in the recognition of diseases within agriculture.

Asif et al. (2020) evaluated various CNN models for the identification of PLD: AlexNet, VggNet, ResNet, LeNet, and a sequential model specifically intended for the purpose. Their sequential model was then able to achieve a high value of precision, amounting to 97% for the detection. The works identified the prime capability of CNN-based models for image classification. This work points out the ability of sequential models in disease detection for agricultural purposes. Lanjewar et al. (2024) applied several transfer learning models—VGG19, NASNetMobile, and DenseNet169—for PLD detection. They modified each model architecture to improve the accuracy in classification. The modified DenseNet model they proposed gave the highest accuracy of 99%. The three backbones span a range of capacity, from the parameter-heavy VGG19, through the mobile-oriented NASNetMobile, to the densely connected DenseNet169. That the densely connected variant proves strongest is consistent with feature reuse being advantageous when the discriminative evidence consists of small lesions whose low-level colour and edge cues must remain accessible to the deepest layers. The architectural modification is not specified beyond the classification head, however, so the 99% cannot be decomposed into the contributions of transfer learning and of the modification itself. Catal Reis and Turk (2024) introduced a novel deep learning model for detection of PLD using depthwise separable convolution and transformer networks, combining DSC with a multi-head attention mechanism. They were able to attain 99.24% accuracy with their MDSCIRNet model, whereas the conventional CNNs such as Xception, MobileNet, and EfficientNetB2 had lower accuracy. DSC and attention can show high effectiveness in increasing the accuracy of plant disease classification, demonstrated by this work. MDSCIRNet is the closest architectural precedent to this study, coupling convolutional feature extraction with multi-head attention, and its margin over Xception, MobileNet and EfficientNetB2 confirms the pairing is beneficial. Evaluation is nevertheless confined to PlantVillage, and the full transformer stack is retained over the entire feature map, so the accuracy is obtained without the parameter budget or environmental variability that edge deployment imposes.

Gurusamy (Gurusamy et al., 2023) proposed a CNN model for automatic detection and identification of PLD. The highest accuracy achieved by this model was 98.1% on the PlantVillage dataset. This work also demonstrated the effectiveness of CNNs for automatic detection of disease and showed further studies towards its extension to other crops. Kotwal et al. (2024) proposed a hybrid model by combining UNet segmentation technique with EfficientNet for plant leaf disease classification. The weights of the UNet model were optimized using the Hunter-Prey Optimization (Hunt-PO) algorithm. The performance achieved by their model was an accuracy of 99.91%. This hybrid model presented how segmentation, feature extraction, and classification techniques go together in an effective manner for plant disease detection. This is the highest figure among the reviewed studies, but also the most dependent on favourable conditions, combining UNet segmentation, an EfficientNet backbone and metaheuristic optimisation on PlantVillage alone. Accuracies above 99.9% on a single controlled dataset indicate benchmark saturation rather than solved difficulty, and the added stages increase training cost and inference latency in a manner the authors do not quantify. Alzakari et al. (2024) reported early detection of potato diseases using the CNN–long short-term memory (LSTM) model. Due to the presence of a powerful feature extraction capability of CNN and strong processing of sequences of LSTMs, the proposed model reached 97.1% accuracy. This hybrid architecture has the potential to improve early disease detection crucial for minimizing losses in crops. **Table 2** presents some methods implemented over recent years.

**Table 2:** Various methods for potato leaf disease detection and classification, with the baseline or competing models against which each result was reported.

Ref.	Year	Method/architecture	Dataset	Baselines Compared	Results
(Rozaqi and Sunyoto, 2020)	2020	Custom CNN for PLD detection	PlantVillage	Not reported	97% training, 92% validation accuracy
(Asif et al., 2020)	2020	Custom sequential CNN, compared against standard backbones	PlantVillage	AlexNet, VggNet, ResNet, LeNet	97% precision
(Khalifa et al., 2021)	2021	14-layer CNN with data augmentation	Custom (expanded 1,722 to 9,822 images)	Previously published results; not retrained	98% accuracy
(Rashid et al., 2021)	2021	Multi-level YOLOv5 segmentation + CNN	PlantVillage	Existing methods, not individually iden- tified	99.75% accuracy
(Singh and Kaur, 2021)	2021	K-means segmentation + multi-class SVM	PlantVillage	Not reported	95.99% accuracy
(Hasan et al., 2021)	2021	CNN comparison; ResNet best per-former	Custom	AlexNet, GoogLeNet	ResNet best; permodel figures not reported
(Chen et al., 2022)	2022	Lightweight CNN with attention (MobOca Net, MobileNet-V2)	PlantVillage	General-purpose CNN backbones	97.73% accuracy
(Ghosh et al., 2023)	2023	CNN comparison; VGG19 best per-former	PlantVillage	DenseNet121, ResNet50	VGG19 best; figures not reported
(Gurusamy et al., 2023)	2023	Custom CNN for automatic disease detection	PlantVillage	Not reported	98.1% accuracy
(Lanjewar et al., 2024)	2024	Transfer learning with modified DenseNet169	Potato Leaf Disease Dataset	VGG19, NASNetMo- bile	99% accuracy
(Catal Reis and Turk, 2024)	2024	DSC + transformer networks (MDSCIRNet)	PlantVillage	Xception, MobileNet, EfficientNetB2	99.24% accuracy
(Kotwal et al., 2024)	2024	Hybrid UNet + EfficientNet with HuntPO optimization	PlantVillage	Not reported	99.91% accuracy
(Alzakari et al., 2024)	2024	CNN-LSTM hybrid	Disease With Weather	Not reported	97.1% accuracy
(Sinamenye et al., 2025)	2025	Hybrid CNN-ViT (EfficientNetV2B3 + ViT)	Potato LeafDisease + PlantVillage	Not reported	85.06% and 98.15%
(Sangar and Rajasekar, 2025)	2025	EfficientNet-LITE + KE-SVM (CBAM, 1D-LBP)	Uncontrolled + PlantVillage	Backbone without KE-SVM (79.38%, 99.07%)	87.82% and 99.54%
(Bajpai and Sahu, 2026)	2026	Ghost convolutions + CBAM + Lite ViT	Field-captured potato leaf dataset	Not reported	99.67% accuracy
(Benbakreti et al., 2026)	2026	ViT vs. CNN comparative study	Kaggle potato leaf (2,152 images)	InceptionV3, DenseNet121, VGG16	97.68% (ViT best)

Recent work has continued to push PLD detection toward hybrid and attention-driven architectures. A combined CNN/transformer-based EfficientNetV2B3+ViT structure utilized parallel extraction of both CNN local texture features and transformer global context, along with dropout-based concatenation, and demonstrated 85.06% on the challenging, uncontrolled-environment potato leaf disease dataset and 98.15% on the Plant Village dataset (Sinamenye et al., 2025). The 13-point spread for a single model quantifies how much of the accuracy reported elsewhere depends on controlled acquisition, and shows that combining convolutional and transformer branches is not by itself sufficient for field robustness without preprocessing designed to stabilise illumination and colour. Focusing on reducing computation for deployment, a two-stage Ghost-CBAM-LiteViT combined efficient Ghost convolutions, spatial and channel attention with CBAMs, and inexpensive Lite ViT blocks for efficient modeling of context, achieved a test accuracy of 99.67% with drastic reduction in computational requirements for application in smart-agriculture environments (Bajpai and Sahu, 2026). Vision Transformer performance was evaluated against three popular CNNs (InceptionV3, DenseNet121, VGG16) and was shown to significantly outperform the CNNs on a 2,152 image Kaggle dataset of potato leaves for both early and late blight detection, achieving a test accuracy of 97.68% (Benbakreti et al., 2026). Lastly, a Kernel-Ensemble SVM approach coupled with the optimization of an EfficientNet-LITE backbone, augmented with channel attention and 1-D LBP local texture features, combined four separate SVM kernel types into an ensemble. This resulted in improved test accuracy from 79.38% to 87.82% and 99.07% to 99.54% respectively on both the uncontrolled-environment potato disease dataset and Plant Village, demonstrating the ability of classic classifiers to assist with the generalization of deep features for a challenging, real-world task (Sangar and Rajasekar, 2025). A custom CNN trained on 16,000 PlantVillage images and validated on 1,000 manually collected field images across five potato leaf conditions further illustrated the value of large-scale augmentation and domain-specific validation for generalization beyond laboratory data (Mulugeta et al., 2025).

Read together, these studies fall into three groups. Handcrafted pipelines combining segmentation with conventional classifiers (Suttapakti and Bunpeng, 2019; Sharma et al., 2021; Singh and Kaur, 2021) plateau between 92.9% and 95.99%, since the colour-based segmentation they depend on is itself sensitive to the illumination variability it must handle. Single-backbone CNN and transfer-learning approaches (Asif et al., 2020; Rozaqi and Sunyoto, 2020; Hasan et al., 2021; Khalifa et al., 2021; Ghosh et al., 2023; Gurusamy et al., 2023; Lanjewar et al., 2024) recover four to six points over that plateau and cluster between 97% and 99%, but capture either local texture or global context alone. Hybrid and attentiondriven architectures (Catal Reis and Turk, 2024; Kotwal et al., 2024; Sangar and Rajasekar, 2025; Sinamenye et al., 2025; Bajpai and Sahu, 2026) report the highest figures, up to 99.91%.

Two observations follow. First, these accuracies are not mutually comparable, as most are obtained on PlantVillage, a saturated benchmark whose uniform backgrounds and controlled illumination make differences of a few tenths of a percentage point uninformative about field behaviour. The evidence is internal to the reviewed literature: the same hybrid CNN–ViT model falls from 98.15% to 85.06% on uncontrolled imagery (Sinamenye et al., 2025), and an ensemble approach records 99.54% and 87.82% across the same pair of datasets (Sangar and Rajasekar, 2025). Second, environmental variability is treated as something to remove before classification, by segmenting the background away (Rashid et al., 2021; Kotwal et al., 2024) or applying generic enhancement, rather than as a property the representation should tolerate; CLAHE, Retinex and RGB-to-HSV each address contrast, illumination or chrominance in isolation. These observations, with the cost incurred by the leading hybrids, define the gap addressed here: a model combining local and global feature extraction, preceded by preprocessing designed for environmental robustness, validated on field-acquired as well as controlled imagery, and small enough for edge deployment.

## 3 Materials and methods

**Figure 1:**
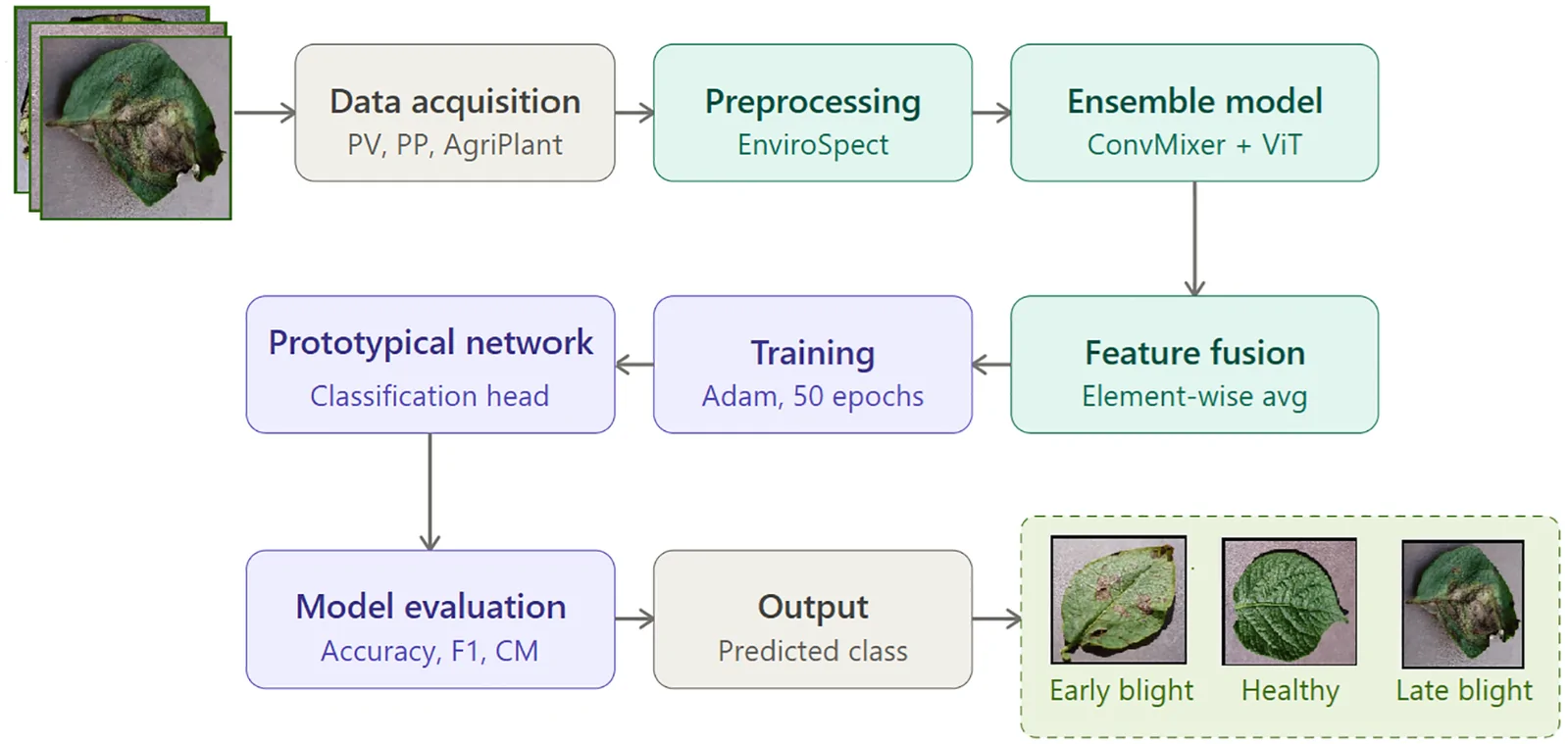
A systematic flow diagram of a proposed ConvMixer-ViT system.

The proposed study integrates the ConvMixer with the model Vision Transformer for developing an ensemble system that would classify PLD through a prototypical network classification approach. It classifies leaf images into three categories: healthy, early blight, and late blight. In the present study, ConvMixer is employed to extract very significant local features of the leaf images, while the Vision Transformer acquires global dependencies through self-attention. Both models are intended to contribute complementary information to the feature representation, and their outputs are combined by elementwise averaging prior to classification. Instead of relying solely on a softmax classifier, the framework uses a prototypical network head that classifies samples by similarity to learned class prototypes. The outputs of the ensemble model are fed into a feature comparison module where class prototypes are created from a few labeled examples. The prototypes and new input samples are further computed for the distance by any similarity metric, for instance, Euclidean distance. The system then categorizes the new images by finding the class with the nearest prototype, thereby increasing the model’s ability to generalize to unseen data with only a few training examples. This prototype based classification approach would make the PLD classification system robust and flexible by establishing decisions on the basis of geometry of the feature space learned. All the steps discussed above is represented in the form of a systematic flow diagram (**Figure 1**).

**Figure 2:**
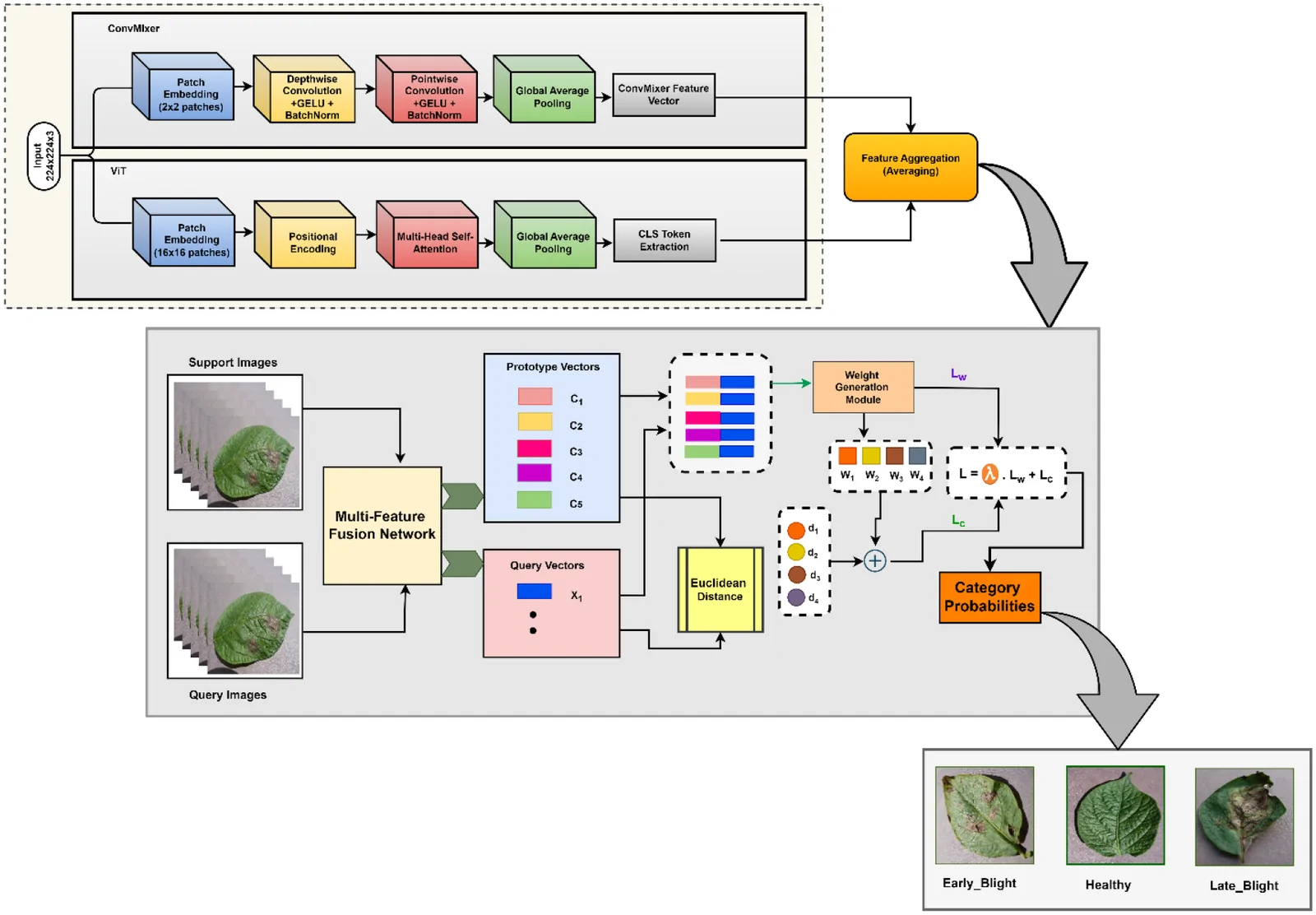
Overall architecture of the proposed ConvMixer-ViT ensemble with prototypical network classification head.

Since the overall architecture is closely tied to the methodology, it is presented here alongside the systematic flow diagram. **Figure 2** depicts the complete ConvMixer–ViT ensemble with the prototypical network classification head. An input leaf image is processed in parallel by two complementary branches: the ConvMixer branch, which uses patch embedding followed by depthwise separable convolutions to capture local, lesionscale features, and the Vision Transformer (ViT) branch, which uses patch embedding, positional encoding, and multi-head self-attention to model long-range global dependencies across the leaf surface. The feature representations produced by the two branches are combined through an element-wise averaging step to form a single fused representation. This fused representation is then passed to the prototypical network head, which forms class prototypes and assigns each query image to the class of the nearest prototype based on Euclidean distance, yielding the final prediction among the healthy, early blight, and late blight categories. The detailed formulation of each component is provided in the following subsections.

### 3.1 Data acquisition

**Figure 3:**
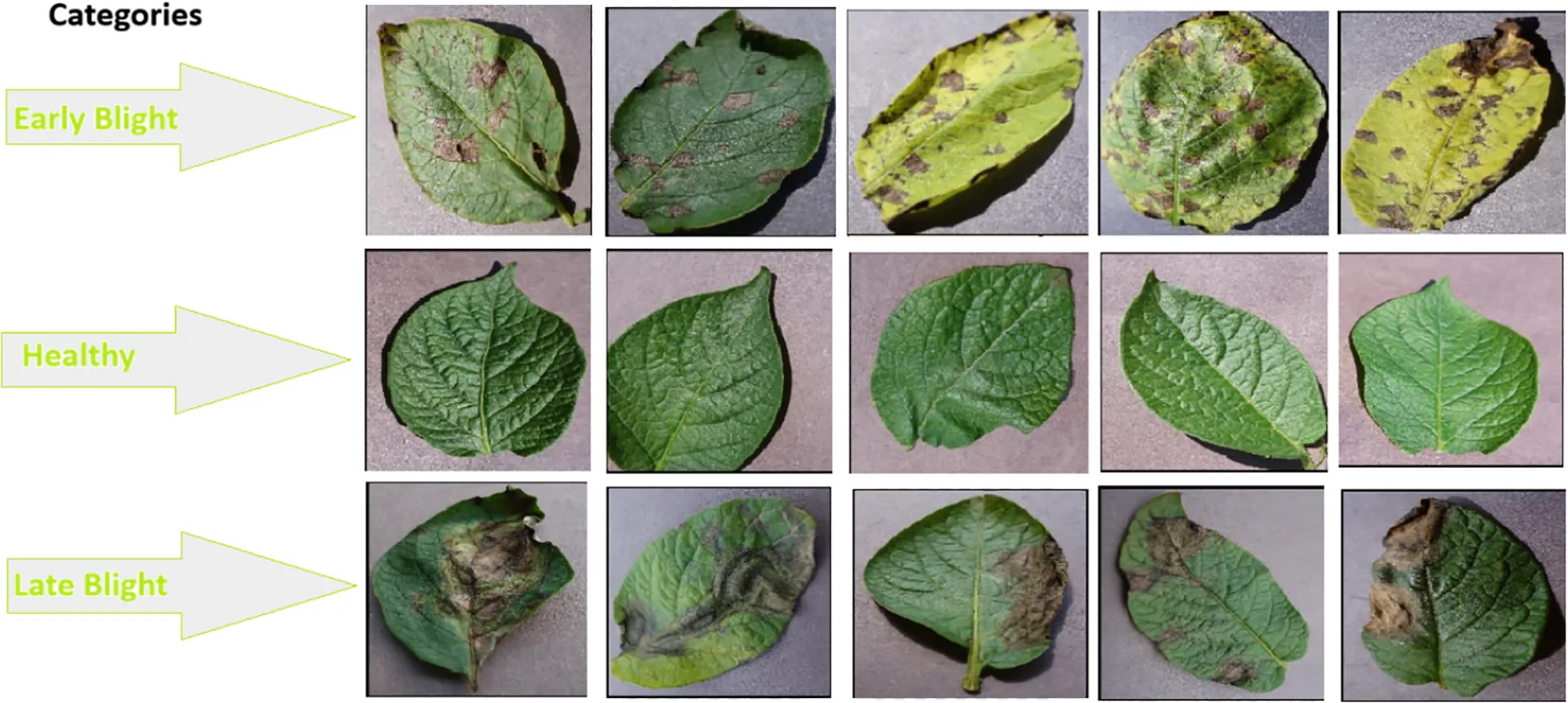
PLD classes are varied, ranging from healthy leaf, early blight, to late blight. Based on the image, one will be able to observe the variation between a healthy and infected leaf, hence enhancing the model to classify them.

For the data collection process, images were collected from 3 separate sources, including PlantVillage (PV) Potato Leaf Dataset, Potato Plant Disease (PP) Dataset (Saleem et al., 2024) and AgriPlant (Private) Dataset. Data from both publicly available benchmarks like the PlantVillage (PV) and Potato Plant Disease (PP) were collected from Kaggle’s data repository and contains 2152 high-quality images with consistent lighting and backgrounds for better feature detection. The AgriPlant dataset is a custom and private dataset, the images in this set have been taken in fields under natural conditions so that it contained variations in illumination, backgrounds, and orientation. The images of both these datasets were then pre-processed using features such as image resolution, illumination, contrast, and color balance. The resolution of all the collected images are resized into 700 × 600 pixels based on the input requirement for any deep learning model, and this is also the very important technique for making the detection of PLD more efficient using computer vision and deep learning. The variety of potato leaf image datasets is illustrated in **Figure 3**. Some of the data collections which are used to produce training and testing sets are tabulated in **Table 3**.

**Table 3:** Summary of the ConvMixer-ViT training and test datasets.

Datasets	No. of classes	Images size	Total images	Notes
PV Dataset (Saleem et al., 2024)	3	700 × 600	2,152	Public benchmark
PP Dataset (Saleem et al., 2024)	3	700 × 600	2,152	Public benchmark
AgriPlant Dataset (Private)	3	700 × 600	1,000	Custom, infield images

To clarify the dataset partitioning strategy, all images were randomly divided into training and testing subsets using a 70%/30% split for each dataset. **Table 4** reports the resulting number of images per subset, together with the overall totals across the three datasets.

**Table 4:** Dataset partitioning for training and testing (70%/30% split).

Dataset	Total images	Training (70%)	Testing (30%)
Plant Village (PV)	2,152	1,506	646
Potato Plants (PP)	2,152	1,506	646
AgriPlant (Private)	1,000	700	300
**Total**	**5,304**	**3,712**	**1,592**

### 3.2 Data preprocessing

The key step in agricultural disease detection is image processing, as it directly influences the quality of features extracted for classification. There exist a number of tested methods of image improvement under various illumination and environmental conditions like CLAHE, Retinex-based algorithms, and RGB-to-HSV conversion.

**Figure 4:**
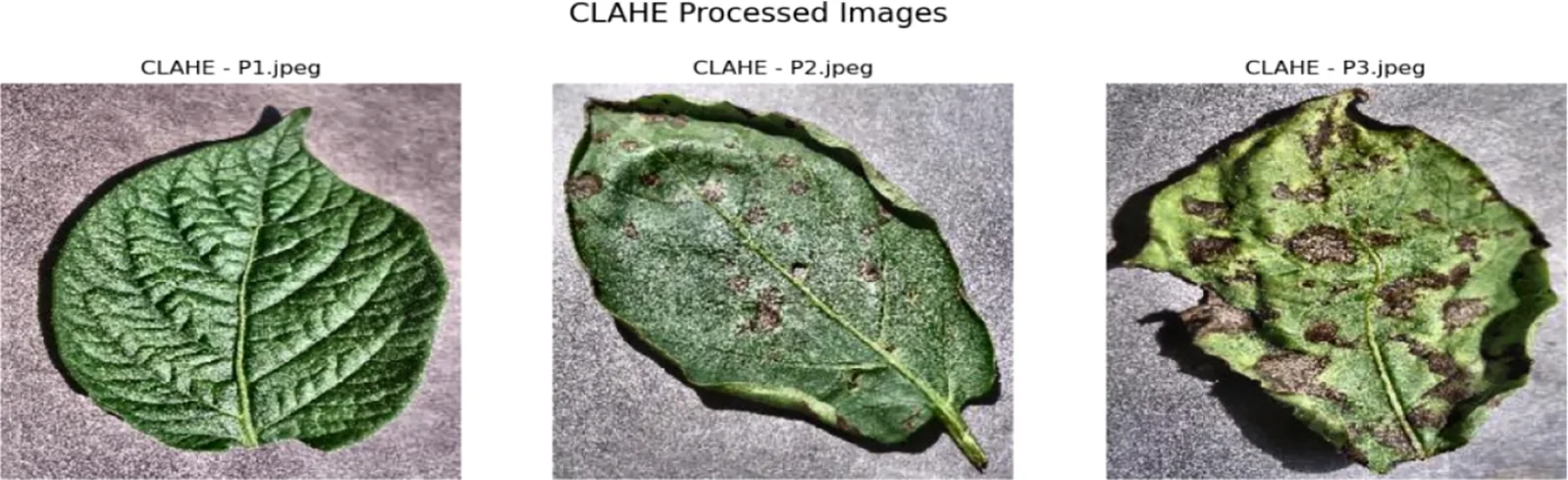
Potato leaf images before and after CLAHE preprocessing.

Contrast Limited Adaptive Histogram Equalization (CLAHE) is often utilized to increase local contrast in an image by splitting the image into small regions and then using histogram equalization for each region. CLAHE works well to enhance local features and reduce global variations in brightness but can lead to noise enhancement and may not selectively enhance color features such as those of significance to plant health, i.e., chlorophyll-filled areas of importance in the detection of diseases. Visual comparison of the CLAHE effect on the potato leaf image, as shown in **Figure 4**, showed that the detail in the leaf texture, lesions, and symptoms of the potato leaf were easier to observe as contrast increased.

**Figure 5:**
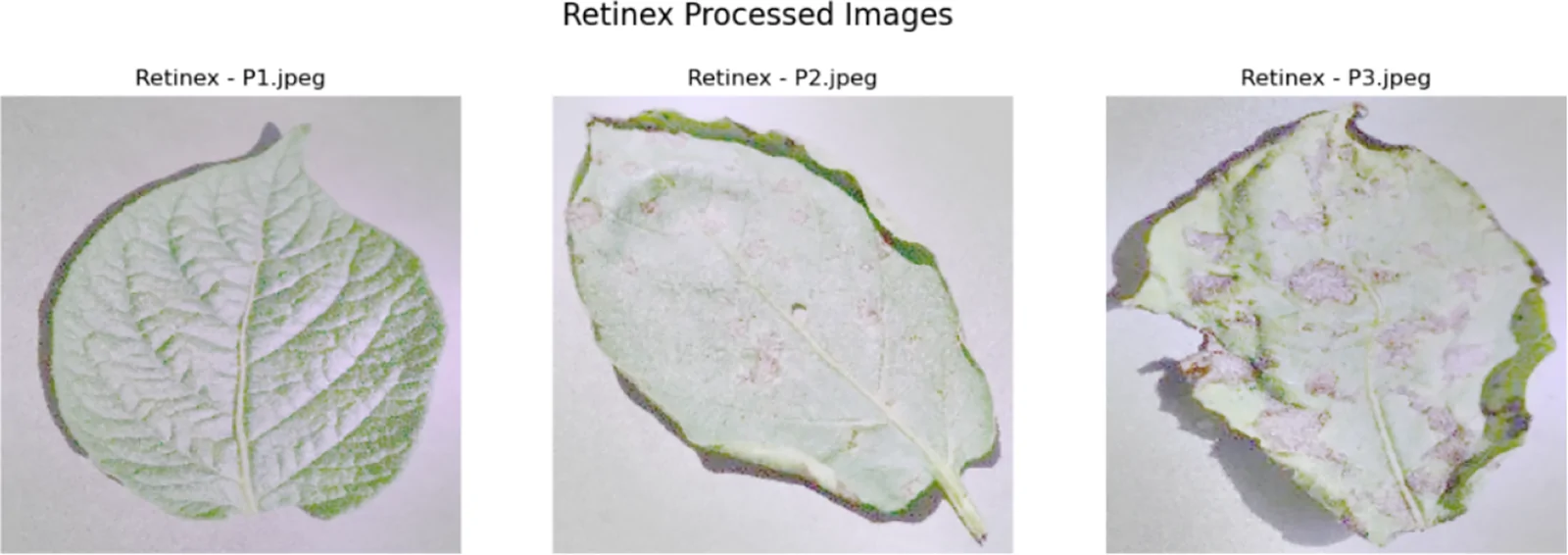
Retinex preprocessing on potato leaf images, normalizing illumination differences.

The Retinex based approaches try to imitate the color constancy mechanism of the human visual system under changing illuminations. By estimating the illuminationindependent portion of the scene. Leads to normalized illumination images. The color constancy is provided by the Retinex processing. While improving the visibility in poor illuminations, the resultant color constancy image tends to contain color artifacts or unrealistically toned image, and the color features of the disease may be marred. **Figure 5** shows the result of Retinex processing on the potato leaf image. The color constancy under illumination normalization improves the visibility, while changing the original color tone values which may be important for diagnosing the disease.

**Figure 6:**
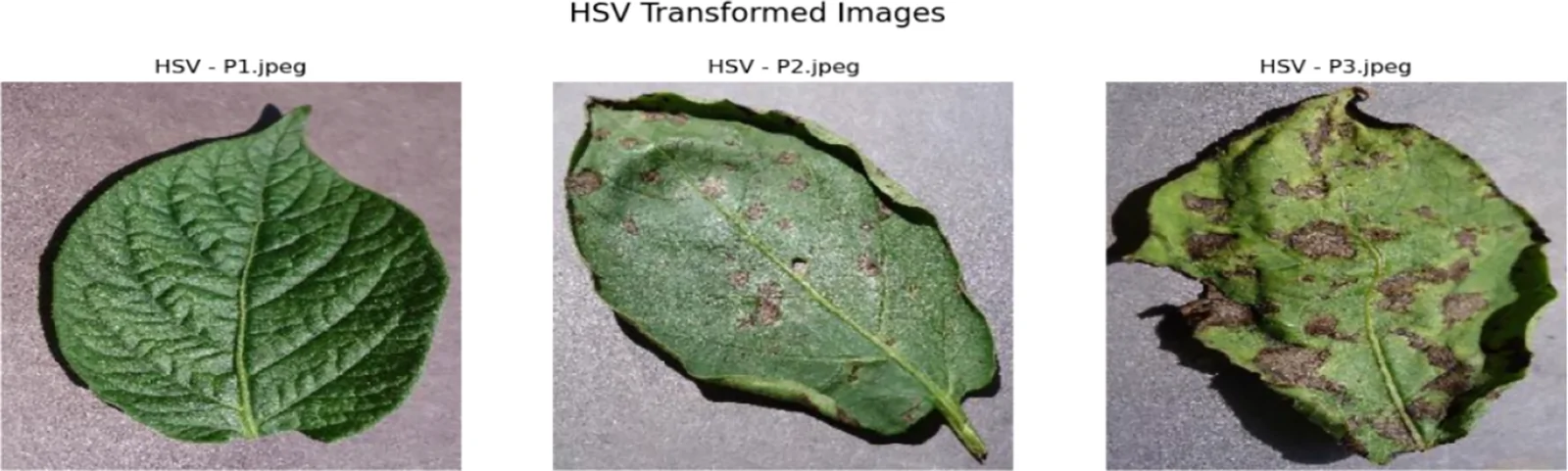
HSV-transformed potato leaf images showing hue-based separation of healthy and diseased regions.

In some applications RGB to HSV transforms are extremely popular. This application concerns segregating chrominance information (Hue & Saturation) from Luminance information (Value or Intensity) such that feature extraction can be more reliably carried out, especially in respect of vegetation. The hue component in HSV can be particularly useful for distinguishing the color of healthy tissue from that of diseased tissue. However, HSV space remains heavily lighting-dependent; it may not effectively normalize luminance changes, thereby providing different feature sets in the same environment. The results of converting the potato leaf image from RGB to HSV with hue-based segmentation can be seen in **Figure 6**. Hue-based segmentation can differentiate disease-related features; however, it can still be affected by lighting.

However, the dedicated process discussed above, called EnviroSpect, is proposed and devised in this paper to localize the most important features of potato leaf images and to provide improved detection and classification under difficult environmental and weather conditions. EnviroSpect transforms an RGB image into a new color space and extracts features based on intensities, saturation, and hue separately, with greater flexibility and precision. With such a conversion, the new colors remain distinguishable despite changes in illumination intensity or a noisy background. After the transformation, the color components are separated to release characteristic information; the least significant or noisy areas are discarded. Thus, the proposed method can better overcome adverse or inconsistent conditions. With modification, the characteristic features of objects are emphasized, and normalization can make images from different weather conditions and varying illumination roughly comparable in brightness and contrast.

**Figure 7:**
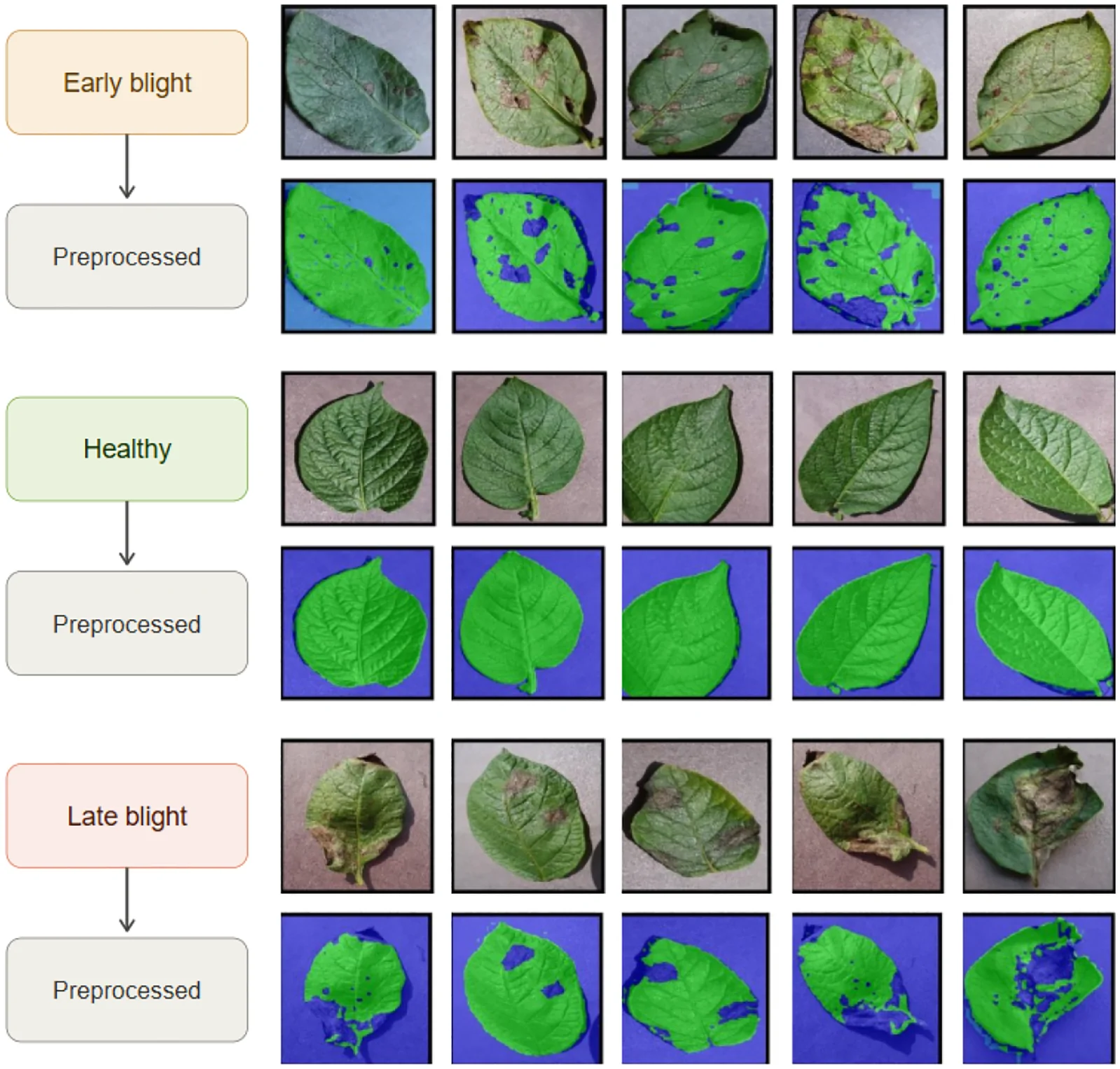
EnviroSpect preprocessing results on potato leaf images under varying environmental conditions.

Compared to those traditional methods, EnviroSpect remaps the hue to emphasis vegetation spectrum features, adds saturation and normalizes the intensity levels. In this way, disease-indicative features (subtle color variations and textural features indicating lesions) remain discoverable and the classification performance are maintained against different environments. The areas displayed in these images after the operation are output after a recomposition or a colormap mapping; and actually it represents the critical regions in disease diagnose. The image shown in **Figure 7** of this work indicates that EnviroSpect provide excellent feature extracting abilities, thus, the analysis and classification methods can be more accurate and reliable.

Compared to other preprocessing methods:

- CLAHE enhances local contrast but often amplifies noise and may not specifically target color features crucial for detecting plant health indicators.
- Retinex-based methods improve illumination consistency but frequently introduce unnatural color artifacts, potentially obscuring subtle disease-specific color changes.
- RGB-to-HSV transformations separate color information effectively but remain sensitive to lighting variations and lack mechanisms for targeted enhancement of disease features.

EnviroSpect surpasses these methods by combining the strengths of color space transformation, adaptive channel enhancement, and context-specific normalization, tailored explicitly for agricultural imaging. It is an efficient and scalable approach to implement effective high-level feature extraction for diseases diagnosis in real agricultural environment in terms of computational efficiency. **Algorithm 1** outlines the whole procedure in EnviroSpect preprocessing pipeline.

Algorithm 1 **EnviroSpect Preprocessing Technique.**

**Require:** RGB potato leaf image *I_RGB_*
**Ensure:** Enhanced image *I_final_* with highlighted disease-relevant features
1: Load input image: *I_RGB_*← cv2.imread(*filepath*)
2: Convert RGB to HSI color space: (*H,S,I*) ← rgb_to_hsi(*I_RGB_*)
3: Remap hue channel to emphasize vegetation: *H_rem_*← remap_hue(*H*)
4: Merge saturation and intensity: *I_sat_*← merge_intensity_saturation(*I,S*)
5: Normalize intensity to [0,255]: *I_norm_*← cv2.normalize(*I_sat_*,0,255,NORM_MINMAX)
6: Overlay remapped hue on enhanced image: *I_final_* ←
merge_hue_on_image(*I_norm_,H_rem_*)
7: Save processed output: cv2.imwrite(*output_path,I_final_*)
8: **return** *I_final_*

EnviroSpect is expected to outperform these methods because of how each treats a leaf image. CLAHE enhances intensity contrast indiscriminately, amplifying background noise while leaving disease-relevant chromatic cues unmodeled. Retinex removes the illumination component for color constancy but can alter the tones that distinguish blight, introducing artifacts. RGB-to-HSV separates chrominance from luminance but applies no targeted enhancement and stays lighting-sensitive. EnviroSpect instead works in an HSI space with disease-aware operations: intensity is normalized for cross-illumination comparability, saturation is enhanced to strengthen healthy-versus-lesion contrast, and hue is remapped toward the vegetation spectrum to emphasize lesion color signatures. Because these per-component operations align with the color and texture cues of foliar disease, disease-discriminative information is preserved while illumination- and backgroundinduced variation is reduced, explaining the larger, more consistent gains for EnviroSpect in **Table 5** and the ablation study.

**Table 5:** Performance comparison of preprocessing techniques.

Preprocessing Method	Accuracy (%)	F1-Score (%)
Raw Images	86.3	85.7
CLAHE	88.9	88.1
Retinex	87.4	86.8
RGB-to-HSV	90.2	89.6
**EnviroSpect**	**99.3**	**99.1**

Bold values indicate the results of the proposed method (EnviroSpect/ConvMixer-ViT).

*I* represents the intensity, which describes the overall brightness of each pixel and is calculated the average of R, G, and B colour channels , as shown in Equation 1:

(1) $$I=\frac{R+G+B}{3}$$

The hue *H* is the color information and can be calculated by:

(2) $$H=\cos^{-1}\left(\frac{\left(R-G\right)+\left(R-B\right)}{2\sqrt{{\left(R-G\right)}^{2}+\left(R-B\right)\left(G-B\right)}}\right)$$

This equation prescribes the angular hue value that may be employed to select particular color ranges and even modify data, like highlighting colors that are related to vegetation or other areas of interest.

To consolidate the preprocessing pipeline, **Table 6** summarizes the methods and their associated parameters, together with the alternative enhancement techniques used for comparison. The proposed EnviroSpect operates in a custom HSI color space, whereas CLAHE, Retinex, and RGB-to-HSV are evaluated as baseline enhancement methods on the same images.

**Table 6:** Summary of preprocessing methods and parameters used in this study.

Stage/method	Parameter	Value/setting
Acquisition resize	Output resolution	700 × 600 pixels
Model-input resize	Network input size	224 × 224 × 3
Normalization	Per-channel mean *µ*	[0.485,0.456,0.406]
Normalization	Per-channel std *σ*	[0.229,0.224,0.225]
Augmentation	Operations	Horizontal/vertical flip, rotation, scaling
EnviroSpect (proposed)		
Color-space transform	Target space	RGB → HSI
Intensity	Definition	*I* = (*R* + *G* + *B*)*/*3
Hue	Definition	Equation 2 angular hue
Hue processing	Operation	Remap to emphasize vegetation
Saturation– intensity	Operation	Merge of *S* and *I* channels
Intensity normalization	Range/method	[0,255], min–max (NORM MINMAX)
Reconstruction	Operation	Overlay remapped hue on normalized image
Baseline enhancement methods (for comparison)		
CLAHE	Role	Local contrast enhancement baseline
Retinex	Role	Illumination-normalization baseline
RGB-to-HSV	Role	Chrominance/luminance separation baseline

### 3.3 Proposed ConvMixer–ViT ensemble architecture with prototypical network head

The proposed architecture in this work integrates both convolution-based and transformerbased models into a robust ensemble for three-class classification of PLD, namely, healthy, early blight, and late blight. This system integrates a ConvMixer model focused on the extraction of local patterns from leaf images (Trockman and Kolter, 2022), while the Vision Transformer will model global dependencies with self-attention mechanisms (Dosovitskiy et al., 2021). These complementary architectures are combined and optimized with a prototypical network classification approach (Snell et al., 2017) in handling such a challenge.

The system processes an input image $X\in {\mathbb{R}}^{H\times W\times 3}$, where *H* and *W* are the height and width (224 × 224 pixels), and 3 refers to the color channels. Preprocessing involves resizing, normalizing, and augmenting the image data. Normalization normalizes the pixel values in a standard range using Equation 3:

(3) $$X_{norm}=\frac{X-\mu }{\sigma }$$

where *µ* = [0.485,0.456,0.406] and *σ* = [0.229,0.224,0.225] are the mean and standard deviation values, respectively.

The ConvMixer model is responsible for the capture of local features and starts by applying the patch embedding transformation. The input image *X_norm_* is passed through a convolutional layer with a patch size of *P* × *P* (where *P* = 2), splitting the image into patches, as defined in Equation 4:

(4) $$Z=\mathrm{Conv}2\text{D}\left(X_{norm},P,P\right)$$

The extracted patches then undergo several depthwise separable convolution layers, each capturing hierarchical local features. The output of the i-th depthwise convolutional layer is given by Equation 5:

(5) $$Z_{i+1}=\sigma \left(\text{BatchNorm}\left(\text{GELU}\left(\mathrm{Conv}2\text{D}\left(Z_{i},k,\text{groups}=d\right)\right)\right)\right)$$

where *σ* denotes the activation function, GELU is the Gaussian error linear unit, *k* is the kernel size, and *d* refers to the dimensionality of the depthwise convolution. These depthwise convolutions are followed by global average pooling, as shown in Equation 6:

(6) $$F_{conv}=\text{GlobalAvgPool}\left(Z_{L}\right)$$

where *L* is the number of ConvMixer layers.

The ViT model works in parallel to capture long-range dependencies between patches. An input image is divided into non-overlapping patches of size 16×16 pixels. Each patch is then flattened and projected to a higher dimension space via a linear transformation, as given in Equation 7:

(7) $$P_{i}=\text{Linear}\left(\text{Flatten}\left(X_{i}\right)\right),\;i=1,\ldots ,N$$

where *N* is the total number of patches. Positional encodings are added to these patches to retain spatial information. These patch embeddings are fed into the transformer encoder, which generates the output via multiple self-attention layers.

The outputs of the ConvMixer *F_conv_* and ViT *F_ViT_* models are aggregated to form a single feature representation. Instead of using simple concatenation, the feature vectors are averaged elementwise, as defined in Equation 8:

(8) $$F_{ensemble}=\frac{1}{2}\left(F_{conv}+F_{ViT}\right)$$

This aggregated feature vector then contains both the local patterns captured by ConvMixer and the global patterns modeled by ViT.

For classification, a prototypical network head is employed since class prototypes are obtained from only a few labeled samples. Let *P_c_*represent the prototype for class *c*, which is computed as the average feature representation of the examples from that class, using Equation 9:

(9) $$P_{c}=\frac{1}{n_{c}}\sum_{i=1}^{n_{c}}F_{i}^{\left(c\right)}$$

where *n_c_*is the number of samples in class *c*, and $F_{i}^{\left(c\right)}$ is the feature vector of the *i*-th sample from class *c*. For a new test image, the model calculates the similarity between the test feature vector *F_test_* and each class prototype using Euclidean distance, as shown in Equation 10:

(10) $$d\left(F_{test},P_{c}\right)=\left\|F_{test}-P_{c}\right\|$$

The class label is assigned based on the minimum distance to the class prototypes.

The ensembled architecture combines the strengths of ConvMixer and Vision Transformer for a very powerful and adaptive general model for PLD classification. In classification, utilization of both local and global features with prototypical network classification allows for greater generalization, especially under minimum labeled data points. This would therefore offer an easy avenue for application in practical agricultural disease monitoring and management. The overall architecture is illustrated earlier in **Figure 2**, which is presented alongside the systematic flow diagram in this section, while **Algorithm 2** provides the detailed step-by-step elaboration of the proposed methodology.

Algorithm 2

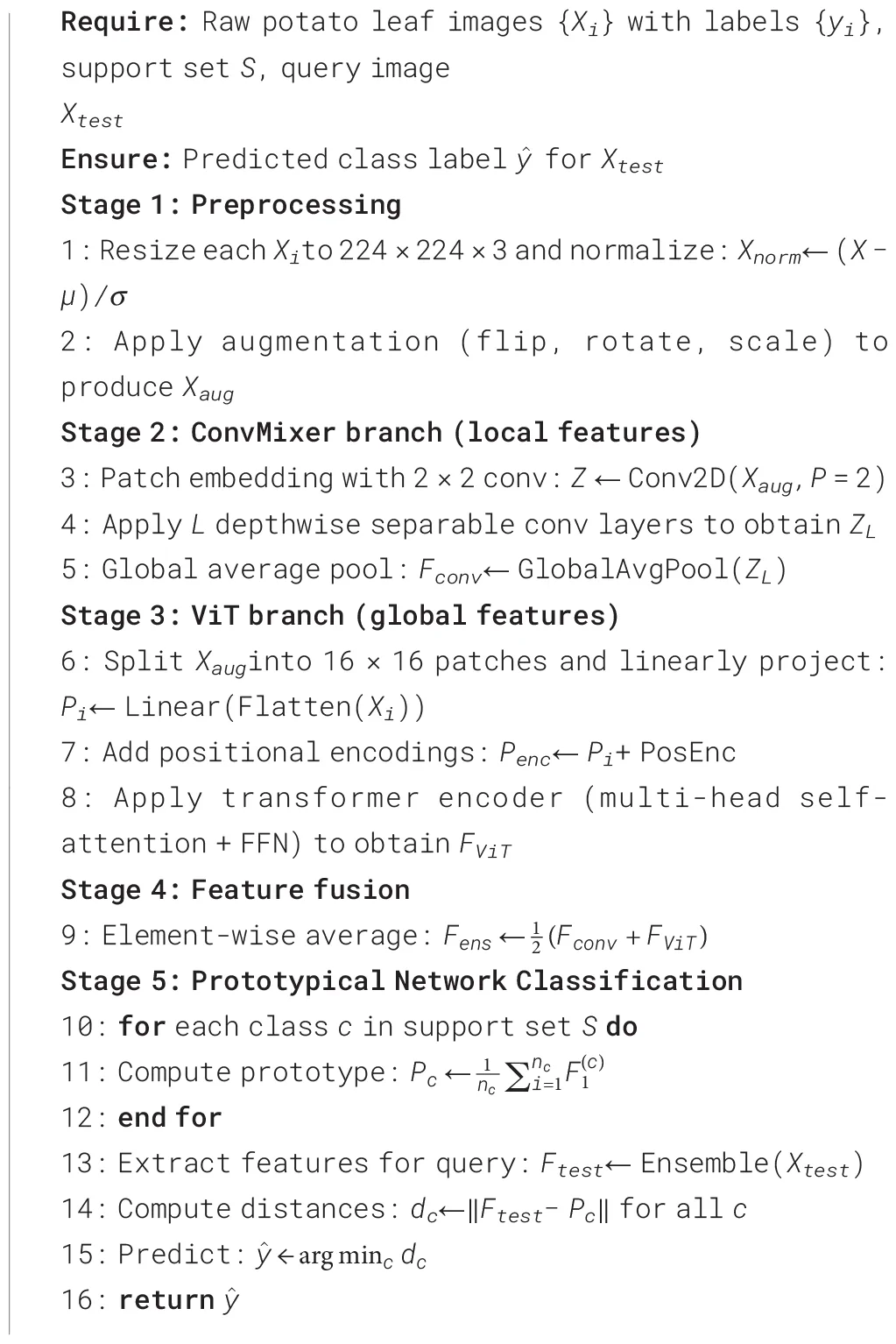

### 3.4 Experimental setup and evaluation metrics

This subsection summarizes the experimental environment, the training parameter settings, and the formulas of the evaluation metrics used throughout the paper. All experiments were implemented in Python 3.10 with PyTorch 2.1 and executed on a workstation equipped with an NVIDIA RTX 3090 GPU (24 GB video random access memory, VRAM), an Intel Core i7-12700K central processing unit (CPU), 32 GB of system random access memory (RAM), and Ubuntu 22.04 long-term support (LTS). The models were optimized using the Adam optimizer with a learning rate of 1 × 10^−4^ and a batch size of 16. Training was run for at most 50 epochs with early stopping when no improvement was observed for 5 consecutive epochs, using a 70%/30% train–test split and an input image size of 224 × 224 × 3. The main hyperparameters and environment settings are summarized in **Table 7**.

**Table 7:** Experimental environment and parameter settings.

Setting	Value
Programming language	Python 3.10
Deep learning framework	PyTorch 2.1
GPU	NVIDIA RTX 3090 (24 GB VRAM)
CPU	Intel Core i7-12700K
System memory	32 GB RAM
Operating system	Ubuntu 22.04 LTS
Optimizer	Adam
Learning rate	1 × 10^−4^
Batch size	16
Maximum epochs	50 (early stopping, patience 5)
Train–test split	70%/30%
Input image size	224 × 224 × 3

The performance of the proposed framework is evaluated using four standard classification metrics: accuracy, precision, recall, and F1-score. These metrics are computed from the numbers of true positives (TP), true negatives (TN), false positives (FP), and false negatives (FN), and are defined as follows in Equations 11–14:

(11) $$\text{Accuracy}=\frac{TP+TN}{TP+TN+FP+FN}$$

(12) $$\text{Precision}=\frac{TP}{TP+FP}$$

(13) $$\text{Recall}=\frac{TP}{TP+FN}$$

(14) $$\text{F}1-\text{Score}=2\times \frac{Precision\times Recall}{Precision+Recall}$$

For the multi-class setting considered in this study, these metrics are computed over the three classes (healthy, early blight, and late blight).

### 3.5 Experimental design

The evaluation comprises a preliminary preprocessing study, four experiments, and a concluding ablation study. The preprocessing study is independent of the four experiments: it fixes the backbone and varies only the preprocessing method, comparing EnviroSpect against CLAHE, Retinex, RGB-to-HSV and unprocessed images, in order to establish which scheme is carried forward.

Experiment 1 asks whether the hybrid architecture outperforms established alternatives, holding the dataset, preprocessing and training protocol constant while varying only the model, against ResNet18, ConvMixer alone, ViT alone, MobileNetV2 and InceptionV3 on PlantVillage. Experiments 2 to 4 ask instead whether performance is stable across acquisition conditions, applying the identical model and protocol to Potato Plants and PlantVillage, both captured under controlled illumination, and to the field-acquired AgriPlant set. The distinction is one of purpose: Experiment 1 is a controlled architectural comparison with the dataset fixed, whereas Experiments 2 to 4 are generalisation tests with the architecture fixed. Experiment 4 is the most demanding, being the only evaluation on imagery acquired outside laboratory conditions.

The ablation study of **Table 8** then decomposes the framework into its three contributions, the hybrid backbone, EnviroSpect preprocessing and the prototypical network head, by introducing them incrementally. **Table 9** summarises the four experiments.

**Table 8:** Ablation study of the proposed ConvMixer–ViT framework on the PlantVillage dataset. The full model (A5) is highlighted.

ID	Architecture	Enviro spect	PN	Accuracy (%)	Precision (%)	Recall (%)	F1-score (%)
A1	ConvMixer	×	×	86.30	86.00	85.50	85.70
A2	ViT	×	×	88.50	88.10	87.60	87.90
A3	ConvMixer + ViT	×	×	92.70	92.40	91.90	92.10
A4	ConvMixer + ViT	✓	×	97.80	97.60	97.30	97.45
**A5**	**ConvMixer + ViT**	✓	✓	**99.00**	**99.20**	**98.85**	**99.02**

Bold values indicate the results of the proposed method (EnviroSpect/ConvMixer-ViT).

**Table 9:** Summary of the four experiments conducted to evaluate the proposed ConvMixer–ViT framework.

Experiment	Dataset	Objective/analysis	Key outcome
Experiment 1	PlantVillage	Compare the proposed ensemble against five baseline architectures (ResNet18, ConvMixer, ViT, MobileNetV2, InceptionV3) under identical conditions	Proposed ConvMixer– ViT ensemble outperforms all individual baseline models in accuracy
Experiment 2	Potato Plants	Training/validation accuracy and loss analysis	98% validation accuracy
Experiment 3	PlantVillage	Training/validation accuracy curves and confusion matrix	99.0% validation accuracy
Experiment 4	AgriPlant (private, in-field)	Training/validation loss and confusion matrix across the three classes	Per-class accuracies of 99.8% (early blight), 99.7% (healthy), and 99.9% (late blight)

## 4 Results

We trained and evaluated the hybrid ConvMixer–ViT ensemble on a total of 5,304 potato leaf images across three classes (healthy, early blight, and late blight), drawn from the PlantVillage, Potato Plant Disease, and AgriPlant datasets described in Section 3. The dataset composition and partitioning are given in **Tables 3** and **4**, and the training configuration and computing environment in **Table 7**. The optimal checkpoint, identified at epoch 40, achieved classification accuracies of 98% on Potato Plants and 99% on PlantVillage, with per-class F1-scores of 0.992 (healthy), 0.995 (early blight), and 0.993 (late blight). The gains over the individual ConvMixer and ViT baselines are quantified in the ablation study of **Table 8**.

### 4.1 Quantitative comparison of preprocessing techniques

**Figure 8:**
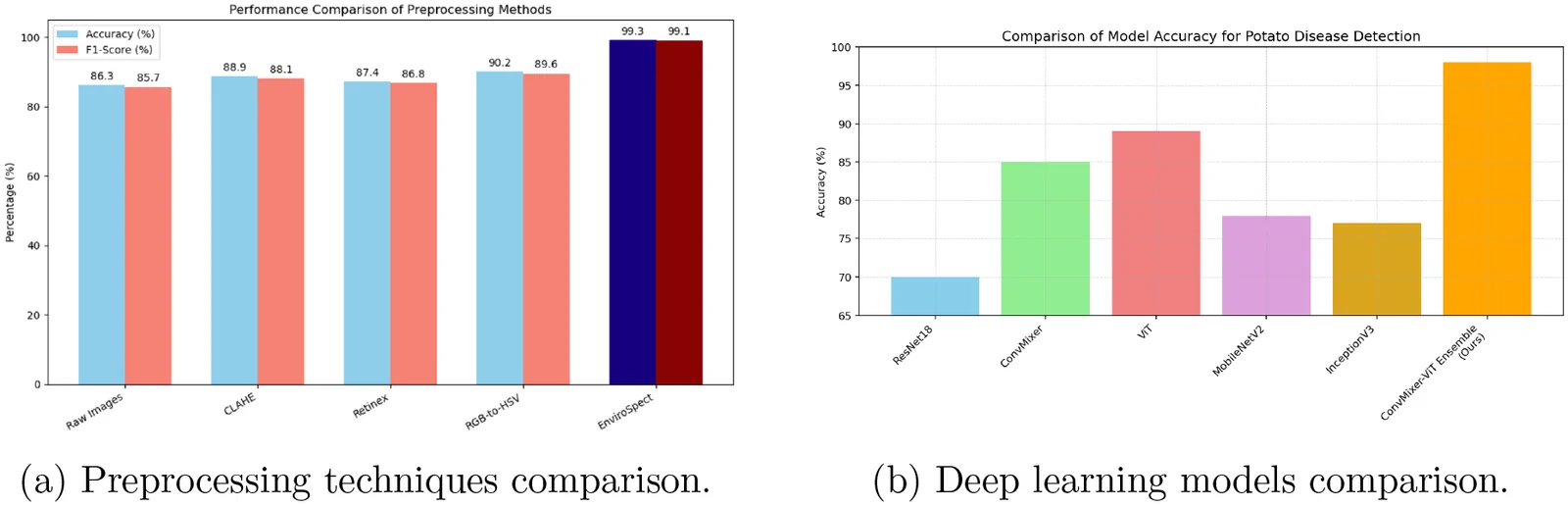
Quantitative comparison: **(a)** classification accuracy and F1-score of ConvMixer with different preprocessing methods (EnviroSpect vs. CLAHE, Retinex, RGBto-HSV, and raw images); **(b)** accuracy comparison across ResNet18, ConvMixer, ViT, MobileNetV2, InceptionV3, and the proposed ConvMixer-ViT ensemble.

In order to substantiate the proposed EnviroSpect preprocessing method, we conducted a quantitative comparative study using various popular image enhancement techniques that are commonly used. Specifically, we compared the classification performance of the ConvMixer model for five different preprocessing techniques applied to images of potato leaves: no preprocessing (raw images), CLAHE, Retinex-based illumination normalization, RGB-to-HSV transformation, and EnviroSpect. Accuracy and F1-score were both employed to evaluate the model performance which are measurements to check how well the model identifies the healthy and diseased leaf tissue. From **Table 5** and **Figure 8a**, we know that with nothing at all, which implies the images are with varied environment and various illumination condition, results in poor performance. By using CLAHE, local contrast is increased, while the accuracy and the F1-score made only moderate improvement. Using Retinex normalization improved the illumination consistence in expense of creating color artifacts, and the classification performance decreased slightly with color artifacts, while converting the image from RGB to HSV helped the color component to separate further giving it some further gain in performance measurements. But EnviroSpect still outrun all of them with greater accuracy and F1-score, that the domain-specific technique used by EnviroSpect by mapping hue, normalizing intensity, and removing noises, proved that this approach can provide better features with varying environment for disease classification.

**Table 5** further shows that EnviroSpect outperforms conventional preprocessing approaches. EnviroSpect proved itself in effectively extracting disease related features and in becoming robust in different environments and lighting conditions.

### 4.2 Experiment 1: comparison with baseline deep learning architectures

The proposed ensemble model is compared to the performance of five deep learning architectures in use. In fact, it refers to models like ResNet18, ConvMixer, ViT, MobileNetV2, and InceptionV3 that were all separately trained for a comparison of results with the proposed ConvMixer-ViT ensemble system. All the models were epoch-trained and under the same conditions for fairness in the test comparison. **Figure 8b** compares various models’ accuracy in percent with the proposed ConvMixer-ViT ensemble. Computational efficiency and inference speed of the ConvMixer-ViT ensemble are also compared with the baseline architectures (discussed in Section 5.2 below). Clearly, from the results depicted in this table, the proposed ensembled system outperformed the individual models on both accuracy-based performance and real-time-based performance. This was because the combination of local feature extraction from ConvMixer with the global attention mechanism from ViT allowed it to achieve a higher classification accuracy. **Figure 8b** presents the accuracy comparison across baseline models and the proposed ensemble, showing the superiority of the proposed system in detecting potato-leaf disease.

### 4.3 Experiment 2: evaluation on the potato plants dataset

**Figure 9:**
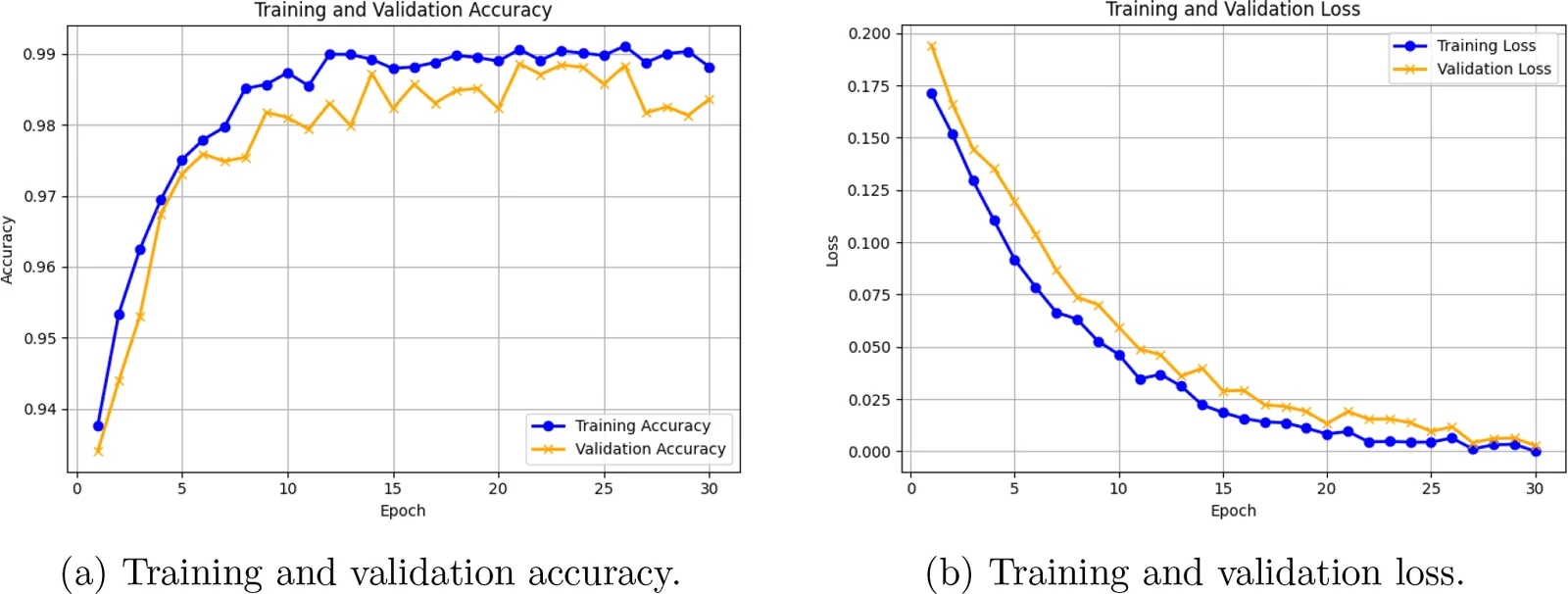
Performance of the ConvMixer-ViT model on the Potato Plants dataset: **(a)** training and validation accuracy, and **(b)** training and validation loss.

In this research study, the performance of our suggested approach was measured on the Potato Plants dataset (Saleem et al., 2024), which was obtained from Kaggle. We examine the loss function in detail as well as the model’s performance on training and validation sets. Accuracy of training and validation obtained by ConvMixer-ViT on this dataset are represented by **Figure 9a** and the corresponding training/validation loss curves are shown in **Figure 9b**. These results show that the model is very capable of classifying the potato plant conditions correctly, as it achieved a high accuracy rate of 98% on the validation set, which demonstrates strong generalization and reliability.

### 4.4 Experiment 3: evaluation on the PlantVillage dataset

**Figure 10:**
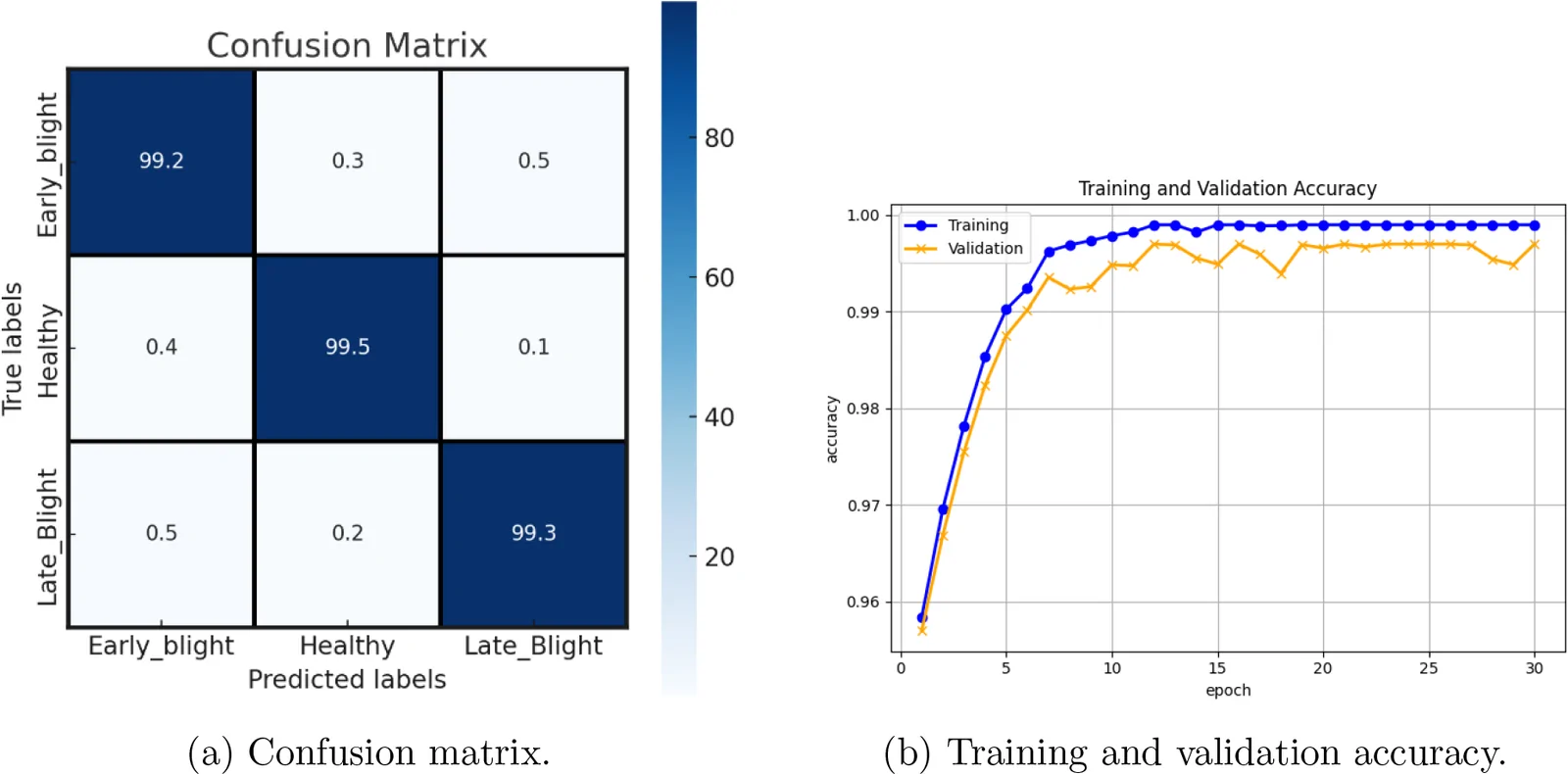
Performance of the ConvMixer-ViT model on the PlantVillage dataset: **(a)** confusion matrix, and **(b)** training and validation accuracy curves.

To justify the performance of our suggested ConvMixer-ViT model, we applied it to the PlantVillage dataset sourced from Kaggle (Saleem et al., 2024). We analyzed the performance of the model on the training and validation sets and observed that the ConvMixer-ViT model was very efficient, as indicated by the loss function trends. **Figure 10b** shows the training and validation accuracies during the procedure, and **Figure 10a** presents the corresponding confusion matrix. This demonstrates that the model is strong in learning and can generalize well. The model reached a classification accuracy of 99.0% on the validation set, therefore showing it is highly effective on the dataset.

### 4.5 Experiment 4: evaluation on the AgriPlant dataset

**Figure 11:**
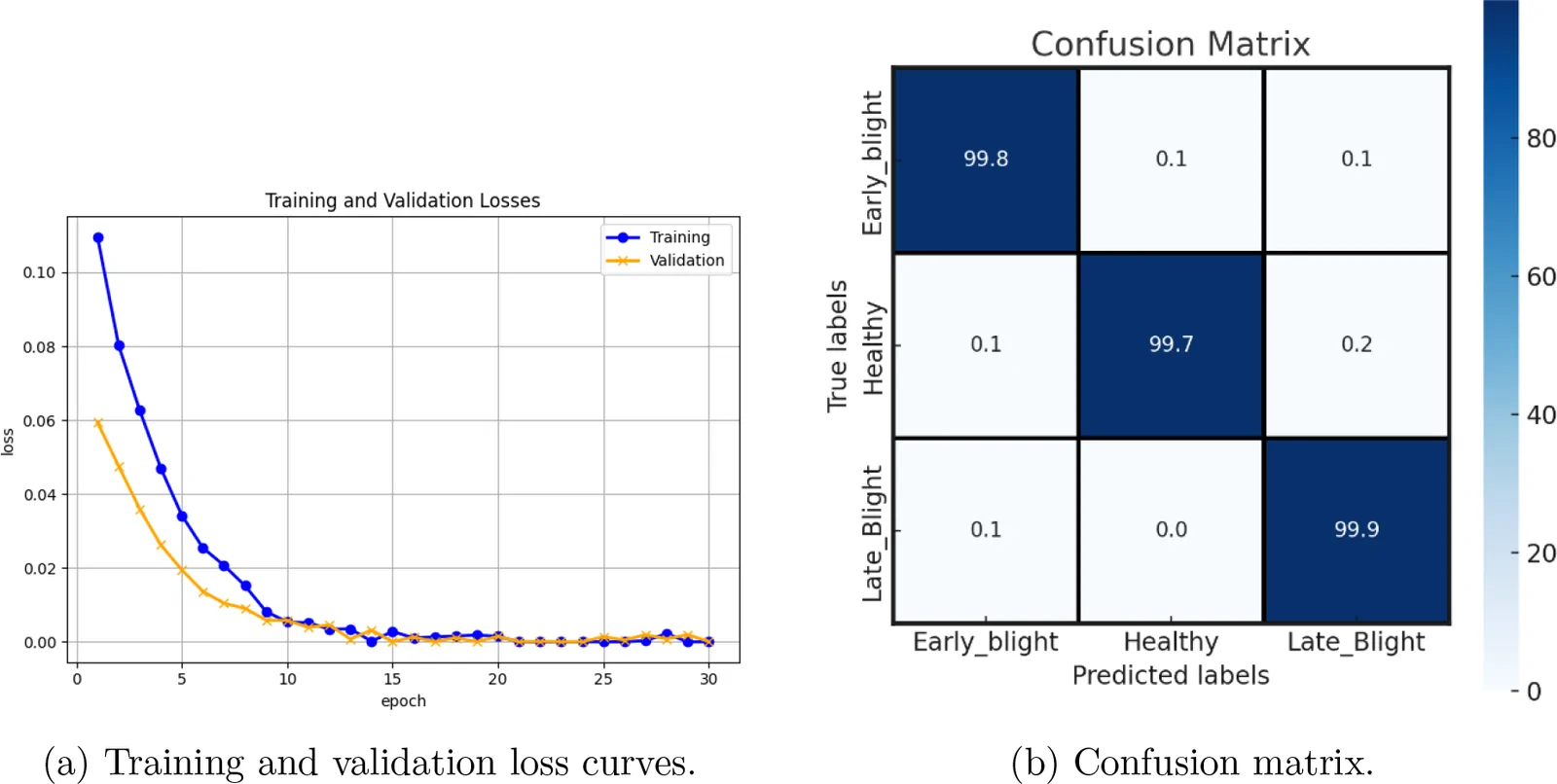
Performance of the ConvMixer-ViT model on the AgriPlant dataset: **(a)** training and validation loss; **(b)** confusion matrix across the three classes.

We tested the ConvMixer-ViT model’s effectiveness by using the private AgriPlant dataset, which contains images of potato leaves. At first, the model’s performance was checked on the training and validation sets. The changes in loss and accuracy showed that ConvMixer-ViT had a strong learning capability and stable convergence throughout the training process. **Figure 11a** presents the training and validation loss curves, while **Figure 11b** shows the corresponding confusion matrix across the three classes. The ConvMixer-ViT model achieved accurate predictions with per-class accuracies of 99.8%, 99.7%, and 99.9% for early blight, healthy, and late blight classes respectively (see **Figure 11b**), demonstrating its high reliability and effectiveness when applied to the AgriPlant dataset.

To provide a concise overview of the experimental evaluation, **Table 9** summarizes the four experiments, including the dataset used, the purpose of each experiment, the main analysis reported, and the key outcome.

### 4.6 Ablation study

To evaluate the individual impact of each module proposed in our framework, we have conducted a series of ablation study on the PlantVillage dataset. We have incrementally introduced ConvMixer, Vision Transformer (ViT), EnviroSpect pre-processing and the prototypical network (PN) classifier. We have trained all configurations under the same setting for 50 epochs using Adam optimizer with 1 × 10^−4^ as the learning rate and 16 as the batch size with 70/30 train–test split. All metrics are averaged over five runs. As can be seen in **Table 8**, after applying ConvMixer directly to the unprocessed image (A1), the achieved accuracy was 86.30% which provides the baseline to show that the local feature extraction could be able to extract the coarse pattern of lesion but sensitive to the change of light source and background. Accuracy increased to 88.50% on replacing ConvMixer with Vision Transformer (A2), which showed better long-range dependence modeling capability over the leaf surface, but performance dropped on non-uniform illumination condition. The combination of these two models as ConvMixer–ViT ensemble (A3) achieved accuracy 92.70% and F1-score 92.10%, which prove the complementing effectiveness of local and global information representation. The highest single gain in accuracy (+5.10%) was obtained after implementing EnviroSpect preprocessing (A4 → 97.80%), which proved the effective use of hue remapping, saturation enhancement, and intensity normalization for environment noise reduction, meanwhile disease-distinguishable information remained intact. The best result with 99.00% accuracy was achieved by adding the prototypical-network-based classification head (A5) to our combined pipeline, resulting in an accuracy, precision of 99.20%, recall of 98.85%, and F1-score of 99.02%. This monotonic increase at each addition demonstrates that all the modules make an actual difference, with the ensemble offering a strong representational baseline and the EnviroSpect significantly improving performance by accounting for environmental differences; the prototypical network head makes decision boundaries sharper on very few labeled data. This corresponds to the published performance (98% and 99% respectively) for Potato Plants and PlantVillage datasets.

## 5 State of the art comparison

The basis of this comparison should be stated explicitly. The figures attributed to competing methods in this section and in **Tables 10** and **11** are those reported by their respective authors. These methods were not re-implemented, retrained or re-evaluated by us under a unified protocol, and were obtained under differing dataset partitions, preprocessing pipelines, augmentation policies, training budgets and, in several cases, differing datasets. Accuracies drawn from separate studies are therefore not strictly commensurable, and the difference between two such values cannot be attributed to the architectures alone.

**Table 10:** State of the art comparison between different methods.

Model	Features	Advantages	Limitations
AgirLeafNet (Saleem et al., 2024)	NASNetMobile + FewShot Learning (FSL), Excess Green Index (ExG)	100% accuracy for potato disease detection, multicrop versatility	Limited robustness to environmental variability
EfficientPNet (Nazir et al., 2023)	EfficientNet-V2, spatialchannel attention, transfer learning, focal loss	98.12% accuracy, resilient to class imbalance and distorted images	Limited to potato-specific application, higher computation for attention mechanism
Attention- Based Lightweight Model (Kasana and Rathore, 2024)	CBAM with pre-trained networks (DenseNet169, MobileNet, VGG16)	Up to 97% accuracy with reduced model complexity, suitable for low-resource deployment	Lower accuracy in varied environmental conditions
ConvMixer- ViT	ConvMixer for local features + Vision Transformer for global context, EnviroSpect preprocessing	High accuracy in varied conditions (98%-99%), balanced local/global feature extraction, adaptable to resource constraints	Higher computational demands than lightweight models

**Table 11:** Performance comparison of state-of-the-art potato disease detection models based on recall, F1 score, and accuracy.

Ref No	Recall	F1 score	Accuracy	Precision
(Saleem et al., 2024)	97%	98%	98%	–
(Nazir et al., 2023)	97.41%	97.65%	98.12%	98.20%
(Kasana and Rathore, 2024)	97.15%	96.99%	97%	95.20%
**ConvMixer-ViT**	**98.85%**	**99.02%**	**99%**	**99.20%**

Bold values indicate the results of the proposed method (EnviroSpect/ConvMixer-ViT).

The comparison is accordingly presented as contextual positioning within the current literature rather than as evidence of superiority. The controlled evidence for our claims lies elsewhere: Experiment 1 compares the proposed ensemble against ResNet18, ConvMixer, ViT, MobileNetV2 and InceptionV3 under identical protocols on a common dataset, and the ablation study of **Table 8** isolates each component. Conclusions regarding the merit of the design rest on those analyses rather than on the cross-study figures below.

Some of the recent approaches for disease diagnosis in potatoes are AgirLeafNet (Saleem et al., 2024), ConvMixer-ViT, EfficientPNet (Nazir et al., 2023), and Attention-based lightweight models (Kasana and Rathore, 2024). AgirLeafNet goes for a combination of NASNetMobile with Few-Shot Learning and the Excess Green Index to achieve the detection of early and late blight diseases in the potato with an outstanding accuracy of 100% classification of potato diseases, and it is also versatile for different types of crops in the field of design consideration. This figure exceeds those obtained by the present framework, and we note it directly rather than omitting it. A reported accuracy of 100% on a controlled benchmark is, however, more readily interpreted as an indication that the benchmark has been saturated than as evidence of perfect discrimination, since it leaves no measurable margin by which any subsequent method could be distinguished, and it is reported without evaluation on field-acquired imagery. In contrast, the ConvMixer-ViT framework is designed to detect PLD by using ConvMixer for capturing local features and the Vision Transformer to model larger contextual information. After using EnviroSpect preprocessing, the performance reached 98% and 99% for the Potato Plants and PlantVillage datasets, respectively. EfficientPNet incorporates spatial-channel attention mechanisms into the EfficientNet-V2 architecture to optimize disease classification accuracy to 98.12% on the PlantVillage dataset. The model is robust to distorted images due to class imbalance by incorporating transfer learning and focal loss. The Attention-based light-weight model leverages the Convolutional Block Attention Module (CBAM) along with pre-trained nets like DenseNet169, MobileNet, and VGG16, which selectively focus on the disease-relevant features to provide high accuracy (97%), reduced model complexity, and hence have the potential for easy deployment on computationally poor devices. While the AgirLeafNet and ConvMixer-ViT prioritize extremely precise multi-crop and potato-specific disease detection, EfficientPNet and the attention-based model mainly focus on achieving efficiency in classification along with robustness against class imbalance and low computational resources. These models, built on top of the PlantVillage dataset, take into account cutting-edge techniques like attention mechanisms and transfer learning, which can be considered a significant technological breakthrough in terms of accuracy, efficiency, and adaptability, and have a massive potential for being implemented in a real-time application in agriculture. The proposed ConvMixer–ViT framework is designed to address several limitations identified in the reviewed literature, in particular the reliance on either local or global features alone, the sensitivity of standard preprocessing to heterogeneous imaging conditions, and the cost of the highest-performing hybrids. Whether this design confers greater robustness than the specific methods discussed above has not been established by direct experiment, however, since those methods were not evaluated under our protocol, and their behaviour under the variability represented in the AgriPlant set is neither reported by their authors nor measured here. What the present results support is narrower: within Experiment 1 the proposed ensemble outperforms the five baselines evaluated under identical conditions, and within the ablation study EnviroSpect accounts for the largest component-wise improvement. To handle this challenge, ConvMixer-ViT uses the EnviroSpect preprocessing technique for normalizing images of environmental conditions in order to retain high accuracy even at the realistic challenging situation. In addition to, the majority of deep learning models being accessed make use of local features or global features only, making them incompetent of representing sufficient information about the leaves’ diseases. This limitation is overcome by marrying the strength of a ConvMixer in extracting local details with the strength of a Vision Transformer in capturing broader contextual information. Such hybrid architecture allows for nuanced and exact classification possible with the ConvMixer-ViT, especially in distinguishing subtle variations between different disease stages. Second, while some of these models are computationally intensive and less adaptive towards resource-constrained settings, ConvMixer-ViT presents a good balance between high performance and efficiency; hence, it is suitable for both high-performance and low-resource settings. Such an advantage promotes the model of ConvMixer-ViT to pragmatic reliable deployment in the field, where variability in environmental conditions and resource limitation normally poses a challenge for conventional models as earlier highlighted in **Table 10**.

ConvMixer-ViT performs favorably among the compared models for potato disease detection owing to its robustness, inclusive feature extraction, adaptability, and high accuracy. While most models suffer from changes in illumination conditions, backgrounds, or quality, ConvMixer-ViT embeds the EnviroSpect preprocessing technique that normalizes images under diverse environmental conditions, making it highly reliable for real-world agricultural applications where such conditions may significantly change. Beyond that, the ConvMixer-ViT model integrates the ConvMixer to learn about the local detail and Vision Transformer for the capture of larger contextual patterns, enabling a better perspective into fine-grained, large-scale features. With that, critical insight into the disease characteristics will be established. In contrast, most of the models focus mainly on either local or global features. For that reason, most of the models will not be able to find the fine difference in the pattern of the diseases. Besides, the ConvMixer-ViT is designed to balance high performance and efficiency well, hence it is a promising candidate for deployment on both high-performance systems and resource-constrained devices with no substantial trade-off in accuracy. The 98%-99% accuracies achieved in the benchmark datasets come very close to the desired accurate, time-and-correctness of disease diagnoses in the domain of agricultural applications. ConvMixer-ViT combines the resilience and adaptability with the accuracy and therefore is more adaptable and reliable for field applications in disease detection of potatoes.

From **Table 11** it can be observed that the proposed ConvMixer–ViT model compares favourably with the state-of-the-art methods listed, subject to the qualification stated above that those values are drawn from the original publications rather than a common protocol. Our model obtained 98.85% Recall, 99.02% F1-Score, 99% Accuracy, and 99.20% Precision, indicating accurate disease detection with few false negatives.

### 5.1 Limitations of the study

A limitation that applies to the comparative analysis itself should be recorded before those of the model are discussed. The competing methods referenced in this paper were not reimplemented or retrained under our preprocessing, training, and evaluation protocol, and their reported figures were obtained on differing dataset partitions and, in several cases, on different datasets. The comparison against published results is therefore indicative rather than conclusive, and the margins separating the proposed framework from those methods in **Tables 10** and **11** should not be interpreted as measured differences in capability. A definitive ranking would require retraining each competing method under a common protocol on a common dataset, including field-acquired imagery, which was beyond the scope of the present study and which we identify as a direction for future work.

Although the developed ConvMixer-ViT framework appears promising for PLD detection, the following shortcomings are identified which can limit the universality of the model in terms of applicability, feasibility of real world usage and extent of use: There are few factors which are needed to satisfy for transferring the model from the laboratory result to the field usage and expand the usage to other crops.

- Controlled Dataset Constraints: The datasets used in this research were collected under controlled conditions. Such datasets are unlikely to be truly representative of real agricultural fields where conditions change due to lighting variations, backgrounds and occlusions. Whilst EnviroSpect preprocessing should enhance robustness, performance is yet to be tested in extreme environments.
- Computational Demands: Significant computational resources are required by the hybrid ConvMixer-ViT model, limiting its deployment in the typically low-power, resource-limited environment of agriculture. Additionally, the model’s emphasis on PLD means its effectiveness against other crop/disease variations has not yet been demonstrated and would necessitate additional adaptation.
- Reliance on Labeled Data: The prototypical network head has an advantage over standard heads in that decisions become more stable; however the system still needs many training samples per class to work. We defer true low-shot scenarios (i.e. 5shot or 10-shot evaluation) to future research and application to less common diseases where sample sizes are few.
- Scalability and Real-Time Deployment: However, the current research did not adequately provide for real-time or large-scale deployments. The considerations required for implementing the model in a field application-speed of inference, power consumption, integration with field-compatible hardware, and etc.-were not fully considered and further investigation in these areas is required.

### 5.2 Computational cost analysis

While the proposed hybrid ConvMixer-ViT model architecture outperforms for detecting PLD, an estimation of its computational cost must also be conducted in order to analyze its feasibility of deployment, particularly in resource constrained agricultural domains. Computational cost, memory requirement, and inference time of an individual ConvMixer, individual ViT, and a hybrid ConvMixer-ViT ensembling model architectures have been compared and estimated. As shown in **Table 12**, In terms of architecture, the low parameters, relatively short computation time makes ConvMixer particularly suitable for deployment to the edge. However, there may be performance compromises as it only focuses on learning global information within the leaf surface. On the contrary, ViT is relatively efficient in capturing long-range dependency, however it has much greater computational cost and larger parameter count for implementing self-attention. The combined architecture presented herein attempts to find an equilibrium such that, in contrast to ConvMixer, it possesses a slightly greater parameter count and computation cost, but significantly lower than ViT, with improved accuracy and robustness. Such compromise indicates the viability of the ConvMixer-ViT model for the efficiency-oriented agriculture environment.

**Table 12:** Computational cost comparison of model architectures.

Model architecture	Parameters (millions)	Inference time (ms/image)	Memory usage (MB)
ConvMixer	3.5	12	150
ViT	21.0	45	420
ConvMixer–ViT Hybrid	8.9	19	240

The results in **Table 12** exhibit the hybrid model of ConvMixer–ViT as a good compromise between model efficiency and computation cost. In terms of computation, it is lighter than a single ViT. However, its feature extraction capability is far better than that of the model without ConvMixer, making it perfect for implementing agriculture in an environment where resources are limited.

We note that the efficiency evidence reported here (parameter count, memory usage, and inference time, including a CPU-only measurement of about 310 ms per image without a GPU) indicates feasibility for low-resource settings, but the framework was developed and trained on a high-end GPU and was not deployed on dedicated embedded platforms such as Raspberry Pi, NVIDIA Jetson, Edge TPU, or mobile devices. The deployment-related statements should therefore be regarded as indicative rather than fully validated on-device, and direct experimental validation on representative edge hardware is left as future work.

### 5.3 Statistical significance analysis

In order to ensure that the noted improvements of the suggested ConvMixer–ViT hybrid model with EnviroSpect preprocessing are reliable and robust, we have done statistical significance testing when comparing its performance to that of baseline preprocessing. We kept track of classification accuracy and F1-score over several experimental runs, and for both metrics, we calculated 99% confidence intervals (CI). Moreover, we carried out paired statistical tests to determine whether performance differences between EnviroSpect and other preprocessing methods were statistically significant. The EnviroSpect method always resulted in higher average accuracy and F1-scores than CLAHE, Retinex, and RGB-to-HSV conversions, as is evident from **Table 13**. The statistically significant improvements have been confirmed by paired *t*-tests (*p<* 0.05), meaning that the high performance of EnviroSpect cannot be due to random data or fluctuation of experimental conditions. These results thus confirm again that EnviroSpect can be considered a reliable and robust method of preprocessing in plant disease detection under variable environmental conditions.

**Table 13:** Statistical significance analysis of preprocessing methods.

Preprocessing method	Accuracy (%) ± CI	F1-score (%) ± CI	p-value vs. enviroSpect
CLAHE	88.9 ± 1.2	88.1 ± 1.4	0.002
Retinex	87.4 ± 1.5	86.8 ± 1.3	0.001
RGB-to-HSV	90.2 ± 1.1	89.6 ± 1.2	0.008
**EnviroSpect**	**99**.**3** ± **0**.**8**	**99**.**1** ± **0**.**9**	—

Bold values indicate the results of the proposed method (EnviroSpect/ConvMixer-ViT).

**Table 13** indicates that EnviroSpect compares exceedingly well to conventional preprocessing methods with statistically significant (*p<* 0.05) increases in accuracy and F1-score (*p<* 0.05), establishing the generalizability and robustness of the suggested method.

## 6 Discussion

Early and late blight rank as the top threats to worldwide potato production. The two pathogens that cause them, respectively, are *A. solani* and *P. infestans*, which induce the diseases by forming spots on leaves, burning them, and finally, making tuber lesions that lead to a decrease in the yield and quality of the crops. The diagnosis of the diseased plants traditionally depends mainly on the visual inspection done by agricultural experts, which, though efficient in a closed environment, turns out to be infeasible for large-scale farming because of its subjectivity, being labor-intensive and time-consuming. These shortcomings indicate the necessity of modern automated techniques that can provide accurate and scalable disease detection in diverse farming conditions. The paper comes up with a solution to such issues with the hybrid deep learning model, the ConvMixer– ViT ensemble, which is a deep learning hybrid model, proposed for the detection and classification of PLD to be improved. The model is empowered by the ConvMixer module to learning fine-grained local features and the Vision Transformer’s (ViT) module to learning global contextual relationships. This hybrid architecture distinguishes between healthy leaves, early blight, and late blight even under diverse environmental conditions. The new model reached high classification accuracies of 98% on the Potato Plants dataset and 99% on the PlantVillage dataset, which are competitive with recent methods in the PLD identification domain.

The novel preprocessing method, EnviroSpect, is one of the significant factors behind the outstanding performance in this case. EnviroSpect is a method that changes the RGB image into a specially designed color space, from which the lightness, the hue, and the saturation can be taken separately. By isolating different color components from an image, the method becomes more sensitive to disease features, for example, color and texture of the lesion, and less sensitive to the environmental factors, i.e., variations in lighting, noise, and interference. EnviroSpect’s robustness ensures that the main visual features are still discernible, thus classification accuracy is increased, even when imaging conditions are non-uniform, which is the case in most real-world agricultural applications. Additionally, the work introduces a prototypical network classification head into the ConvMixer–ViT framework. Rather than relying solely on a softmax output layer, the model forms class prototypes from fused features and classifies new samples by proximity to these prototypes. This provides more stable decision boundaries and a natural pathway to extending the framework to low-shot scenarios in future work.

The ConvMixer–ViT ensemble shows substantial benefits compared to current models like EfficientPNet and AgirLeafNet. EfficientPNet, a combination of EfficientNet-V2 with spatial-channel attention, attains a very high accuracy (98.12%) but is mainly a potato application-oriented model. In addition, it has problems with computational efficiency in environments with limited resources. AgirLeafNet, which fuses NASNetMobile with few-shot learning to achieve almost perfect accuracy, however, has not been able to build a robust environment like the one constructed by the ConvMixer–ViT ensemble, thus its practical application is limited in different agricultural areas. The ConvMixer–ViT hybrid architecture that integrates local and global feature extraction provides a more flexible and dependable way to be used under different field conditions. Besides the potato disease detection, the ConvMixer–ViT ensemble is compelling, which means it can be used in many different ways that will benefit the agricultural sector. Its design can easily be changed for crops such as tomatoes, peppers, and cucumbers that are equally affected by fungal and bacterial diseases. As a result, the model becomes more versatile regarding the number of disease symptoms it can recognize, as it can detect local and global patterns for several plant families. In addition, its use can go much beyond just identifying diseases to encompass pest identification, which is a source of information that can facilitate the implementation of integrated pest management (IPM) strategies by allowing the precise interventions to be undertaken as a result of the early visual sign detection. This can help reduce the excessive use of pesticides, thereby making farming more sustainable.

The EnviroSpect preprocessing method is beneficial for various crop-monitoring applications, including drought, nutritional stress, and air-pollution stress, where it produces visually consistent features across conditions, thereby reducing response time and enabling prompt decision-making. It also offers practical throughput and large-scale processing capability, making it suitable for precision agriculture and for a real-time, effective Internet of Things (IoT)-based agricultural land monitoring system, which would be greatly beneficial for smallholder farmers with very little knowledge of expert agronomists. These are cost-effective methods for disease detection.

The main findings of this study can be summarized as follows. First, the ablation analysis showed a consistent, monotonic improvement in accuracy as each component was added: the single-branch baselines achieved 86.30% (ConvMixer) and 88.50% (ViT), the ConvMixer–ViT ensemble reached 92.70%, the addition of EnviroSpect preprocessing raised accuracy to 97.80%, and the final prototypical network head produced the best result of 99.00%. Among these components, EnviroSpect provided the largest single gain in accuracy (+5.10%), which highlights that environment-robust preprocessing, rather than model capacity alone, is a key driver of performance under variable field conditions. Second, the statistical significance analysis confirmed that the improvements of EnviroSpect over CLAHE, Retinex, and RGB-to-HSV are significant (*p<* 0.05), indicating that the gains are reliable rather than an artifact of experimental fluctuation. Third, the framework attains this accuracy while remaining computationally efficient, using only 8.9 M parameters and requiring approximately 19 ms per image on GPU and about 310 ms per image on a CPU-only setting, which supports its feasibility for resource-constrained deployment. The principal implication of these findings is that combining a hybrid local– global backbone with domain-specific preprocessing and a similarity-based classification head yields an accurate yet efficient solution that is well suited to real agricultural environments and transferable to other crops and imaging conditions.

At the same time, this study has several limitations that motivate future research. The benchmark datasets were largely acquired under controlled conditions and may not fully represent the extreme variability, occlusion, and background clutter of open fields; the hybrid architecture, although lighter than a standalone ViT, still imposes higher computational cost than the most lightweight models; and the prototypical network head, while improving decision stability, still relies on a sufficient number of labeled samples per class, so genuine low-shot performance (for example 5-shot or 10-shot evaluation) has not yet been established. These points are discussed in detail in the Limitations of the Study subsection. Accordingly, future work will focus on validating the framework on larger and more diverse field-acquired datasets, extending the evaluation to true low-shot scenarios and to additional crops and diseases, and optimizing the model for edge deployment through techniques such as pruning, quantization, and knowledge distillation, ultimately integrating it into a mobile-based advisory system for practical use by farmers.

Overall, the ConvMixer–ViT ensemble represents a meaningful advance in AI-based plant disease diagnosis. By combining a hybrid backbone, EnviroSpect preprocessing, and a prototypical network classification head, the framework achieves strong accuracy, robustness, and adaptability. Its adaptability suggests it could be extended to other crops, pests, and abiotic stresses, contributing to higher crop yields and improved global food security. Future work will focus on optimizing the model for edge-device deployment and integrating it into broader agricultural decision-support systems.

In the future, the ConvMixer-ViT framework will be integrated into a mobile-based advisor system that uses image-based disease-detection methods and text-based guidance to manage common potato diseases, thereby increasing usability and practicability.

## 7 Conclusions

This research addresses the problem of accurately detecting PLD under real agricultural conditions, where existing methods struggle with variable illumination, backgrounds, and orientation, confuse visually similar disease stages, and demand resources unavailable on typical field devices. The proposed environment-robust, efficient framework integrates three complementary elements: a hybrid ConvMixer–ViT backbone capturing local and global features, the EnviroSpect preprocessing scheme that preserves diseasediscriminative cues under heterogeneous conditions, and a prototypical network head for stable similarity-based decisions. Its practical significance is a reliable, resource-efficient tool for scalable potato disease monitoring that could be extended to other crops. The proposed model achieved 98% accuracy on the Potato Plants dataset and 99% on the PlantVillage dataset, effectively classifying healthy, early blight, and late blight conditions. Although the findings are encouraging, more investigation is required to confirm applicability to different crops and environments. Future research will focus on increasing dataset variety across geographical areas, incorporating additional environmental variables (e.g., humidity, soil quality), and optimizing the model for mobile and edge deployment.

## Data Availability

The raw data supporting the conclusions of this article will be made available by the authors, without undue reservation.
